# Drug-Free Mesoporous Silica Nanoparticles Enable Suppression of Cancer Metastasis and Confer Survival Advantages to Mice with Tumor Xenografts

**DOI:** 10.1021/acsami.4c16609

**Published:** 2024-10-24

**Authors:** Yu-Tse Lee, Si-Han Wu, Cheng-Hsun Wu, Yu-Han Lin, Cong-Kai Lin, Zih-An Chen, Ting-Chung Sun, Yin-Ju Chen, Peilin Chen, Chung-Yuan Mou, Yi-Ping Chen

**Affiliations:** † Department of Chemistry, National Taiwan University, Taipei 10617, Taiwan; ‡ Graduate Institute of Nanomedicine and Medical Engineering, College of Biomedical Engineering, Taipei Medical University, Taipei 11031, Taiwan; § International Ph.D. Program in Biomedical Engineering, College of Biomedical Engineering, Taipei Medical University, Taipei 11031, Taiwan; ∥ Nano Targeting & Therapy Biopharma Inc., Taipei 10087, Taiwan; ⊥ Graduate Institute of Biomedical Materials & Tissue Engineering, College of Biomedical Engineering, Taipei Medical University, Taipei 11031, Taiwan; # Research Center for Applied Sciences, Academia Sinica, Taipei 11529, Taiwan

**Keywords:** mesoporous silica nanoparticles (MSNs), metastasis, focal adhesion turnover, cell motility, angiogenesis

## Abstract

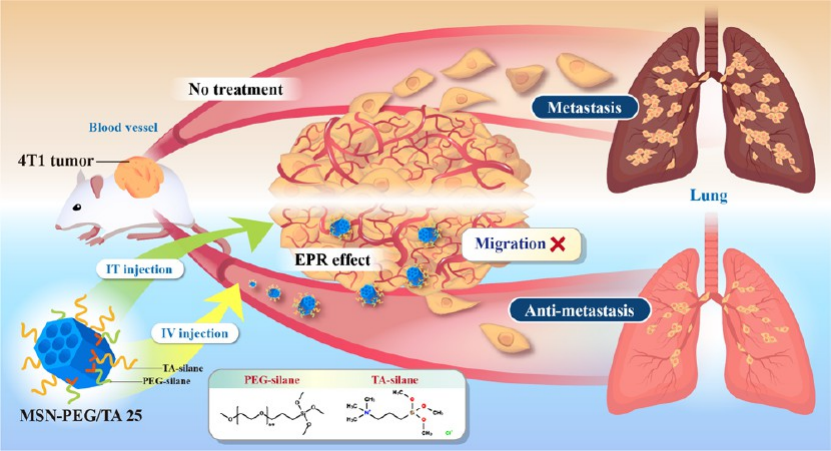

Despite advancements in nanomedicine for drug delivery, many drug-loaded nanoparticles reduce tumor sizes but often fail to prevent metastasis. Mesoporous silica nanoparticles (MSNs) have attracted attention as promising nanocarriers. Here, we demonstrated that MSN-PEG/TA 25, with proper surface modifications, exhibited unique antimetastatic properties. In vivo studies showed that overall tumor metastasis decreased in 4T1 xenografts mice treated with MSN-PEG/TA 25 with a notable reduction in lung tumor metastasis. In vitro assays, including wound-healing, Boyden chamber, tube-formation, and real-time cell analyses, showed that MSN-PEG/TA 25 could modulate cell migration of 4T1 breast cancer cells and interrupt tube formation by human umbilical vein endothelial cells (HUVECs), key factors in suppressing cancer metastasis. The synergistic effect of MSN-PEG/TA 25 combined with liposomal-encapsulated doxorubicin (Lipo-Dox) significantly boosted mouse survival rates, outperforming Lipo-Dox monotherapy. We attributed the improved survival to the antimetastatic capabilities of MSN-PEG/TA 25. Moreover, Dox-loaded MSN-PEG/TA 25 suppressed primary tumors while retaining the antimetastatic effect, thereby enhancing therapeutic outcomes and overall survival. Western blot and qPCR analyses revealed that MSN-PEG/TA 25 interfered with the phosphorylation of ERK, FAK, and paxillin, thus impacting focal adhesion turnover and inhibiting cell motility. Our findings suggest that drug-free MSN-PEG/TA 25 is highly efficient for cancer treatment via suppressing metastatic activity and angiogenesis.

## 1 Introduction

Metastatic disease is the primary cause of death for most cancer patients, rather than the tumor at the primary site.^1^ Current clinical chemotherapeutic treatments are ineffective toward metastasis and can even exacerbate it.^2,3^ Most clinical drug development relies on demonstrating tumor shrinkage without considering metastasis inhibition. Indeed, treating metastatic tumors following conventional therapies is currently a challenge for medical scientists. Therefore, there is an urgent need for novel therapeutic strategies that can simultaneously repress primary tumors and prevent their metastasis.^1^ One of the reasons for not using metastasis as a clinical end-point is that there are difficulties in detecting small metastasis nodules in human patients.^4^ However, technological progress in identifying metastatic sites in patients may produce a paradigm shift toward developing drugs to prevent or treat cancer metastasis.^5^ The emergence of next-generation imaging (NGI) techniques, such as whole-body morphological imaging and diffusion-weighted magnetic resonance imaging,^6^ and various positron emission tomography (PET) tracers together with assistance from deep machine learning^7^ have facilitated moves toward more accurate detection of true metastases.^8^ In August 2021, the US Food and Drug Administration (FDA) announced its finalized guidance on the use of metastasis-free survival (MFS) as an alternative end point for clinical trials, focusing on patients with nonmetastatic castration-resistant prostate cancer (nmCRPC).^9^ This guidance will encourage the development of new therapeutics for treating cancer and preventing metastasis.

One of the most promising approaches for treating metastases is the use of various types of nanoparticles (NPs) to carry drugs with simultaneous antimetastatic effects of NPs.^10−12^ The metastatic cascade promotes the migration of cancer cells from primary to metastatic sites. Modulation of cell migration is one of the responses that occur when cells are treated with some NPs, and it is essential for cancer invasion and extravasation during the metastasis process as documented in recent studies.^13−16^ Metal NPs, such as gold, silver, platinum, titanium, and copper NPs, effectively induce apoptosis in cancer cells, primarily through the generation of reactive oxygen species (ROS), thereby triggering the apoptosis signaling pathway.^17−20^ While previous in vitro studies have provided significant evidence of the antimetastatic effects of NPs, including those without drug loading, by modulating cell signaling pathways in cancer cells, there are still limited investigations into the underlying biological mechanisms in vivo. For instance, Kovacs and colleagues demonstrated gold-core silver-shell (Au@Ag) NPs in antimetastatic and anticancer agent roles, that impacted gene expressions, cancer-associated fibroblast secretory profiles, and tumor cell growth.^21^ Similarly, Arvizo’s group showed that unmodified gold NPs (AuNPs) could inhibit cancer cell proliferation and metastasis by disrupting mitogen-activated protein kinase (MAPK) signaling and reversing epithelial-mesenchymal transition (EMT) in cancer cells.^12^ Zamborlin et al. demonstrated that gold- and copper-based hybrid Nanoarchitectures reduced tumor growth and metastasis progression by altering gene and protein expression of the EMT-related factors.^22^ Moreover, the persistence of noble metals in the human body poses a significant barrier to their clinical translation, despite their promising antimetastatic properties.

Solid NPs mentioned above have little capacity for drug loading and delivery. Such a limitation has produced significant interest in using nontoxic nanoporous inorganic NPs, specifically mesoporous silica NPs (MSNs), due to their excellent capability to deliver cancer drugs to tumors passively.^23,24^ Owing to their attractive attributes, such as biocompatibility, a substantial surface area, ease of functionalization for targeting, adjustable particle sizes, and tunable nanopores, MSNs have found widespread use in nanomedicine for accommodating diverse cancer drugs.^25^ Chen et al. demonstrated that hollow MSNs loaded with an antimetastasis drug (silibinin) could suppress tumor metastasis *in vitro*.^26^ Notably, CuS@mSiO2-polyethylene glycol (PEG) core–shell NPs were shown to inhibit cancer migration by interacting with matrix metalloproteinase (MMP)-2, MMP-9, SRC, and focal adhesion (FA) kinase (FAK) pathways, thereby reducing cell migration.^27^ However, it is not clear in this work which component, Cu or silica, is responsible for the inhibition of cell migration. The therapeutic potential of drug-free MSNs in directly inhibiting tumor metastasis has not been fully explored, yet they hold great promise as novel self-therapeutic NPs. Later research identified MSN’s antitumoral-angiogenesis capability via the ROS-regulated p53 tumor-suppressor pathway. In addition, control of endothelial cell (EC) migration, invasion, and proliferation by *in vitro* drug-free MSNs was reported to be size-dependent.^28^ Although several groups reported that MSNs can suppress cell migration, which could be related to cancer metastasis, most studies were conducted in vitro. A knowledge gap exists between *in vitro* cell migration inhibition and *in vivo* tumor antimetastasis, hampering the development of MSNs as antimetastatic agents.

Recently, we developed a unique design of 25 nm diameter mesoporous silica nanoparticles (MSNs) by tailoring the ratio of short chain polyethylene glycol (PEG) and quaternary amine of N-trimethoxysilylpropyl-N,N,N-trimethylammonium chloride (TA) molecule at a 2:1 ratio (denoted as MSN-PEG/TA 25), which showed promising potential as effective carriers for targeted drug delivery across the blood-brain barrier. This presents a streamlined approach to treat glioblastoma and other brain tumors, even those resistant to standard chemotherapy.^29^ In this work, we found that MSN-PEG/TA 25, even without drug loading, exhibited antimetastatic properties. This exciting finding motivated us to investigate further the effects of MSN-PEG/TA 25 on tumor metastasis, highlighting its significant scientific value in nanomedicine. In this study, we provide evidence that MSN-PEG/TA 25 with no drug loading can hinder cell migration once the NPs are taken up by cells. Moreover, while treatment with MSN-PEG/TA 25 in tumor-bearing mice did not affect the growth of primary tumors, it exhibited a strong inhibitory effect on tumor metastasis. Furthermore, the combination therapy of MSN-PEG/TA 25 and liposome-encapsulated doxorubicin (Lipo-Dox) successfully presented a synergistic effect, resulting in a significant reduction in cancer metastasis, thereby enhancing the overall survival of mice. MSN-PEG/TA 25 acting as nanocarriers for Dox encapsulation effectively inhibited the growth of primary tumors and their metastasis in a 4T1 xenograft mice model, thereby improving therapeutic outcomes and overall survival. Finally, we investigated the molecular pathways affected by cytoskeletal proteins involved in cell migration and angiogenesis.

The studies clearly demonstrate that drug-free MSN-PEG/TA, with its unique structural features and surface modification, holds promise as a therapeutic NP due to its antimetastatic activity. This design, specifically tailored for antimetastasis, is both novel and significant. While it does not inhibit primary tumor growth, given its lack of chemotherapeutic agents, its primary role is in modulating cell migration and angiogenesis rather than serving as a direct therapeutic drug. This function contributes to its antimetastatic properties, making it distinct from conventional chemotherapeutic agents. Our study is the first to demonstrate such effects specifically with MSN-type NPs, emphasizing the unexpected and noteworthy performance of drug-free MSN-PEG/TA.

## 2 Materials and Methods

### 2.1 Chemicals and Reagents

All reagents were obtained from commercial suppliers without further purification. Cetyltrimethylammonium bromide (CTAB, 99%+), tetraethyl orthosilicate (TEOS, 98%), ammonium hydroxide (28–30 wt % in water), (3-aminopropyl)trimethoxysilane (APTMS, 95%), ammonium bicarbonate (99%), glycerol (99%+), and Tween 20 were purchased from Acros Organics (Germany). 2-[Methoxy(polyethyleneoxy)_6–9_propyl]trimethoxysilane, tech-90 (polyethylene glycol (PEG)-silane, M.W. 460–590 g/mol), and N-trimethoxysilylpropyl-N,N,N-trimethylammonium chloride (TA-silane, 50% in methanol) were purchased from Gelest (Morrisville, PA, USA). Rhodamine B isothiocyanate (RITC) was purchased from Cayman Chemical (Ann Arbor, MI, USA). Hydrochloric acid (36.5% ∼ 38.0%) was purchased from J.T Beaker (Radnor, PA, USA). Fetal bovine serum (FBS), penicillin, and streptomycin were purchased from Hyclone Laboratories (Logan, UT, USA). Medium 199 and RPMI 1640 medium were purchased from ThermoFisher Scientific (Waltham, MA, USA). Endothelial cell growth supplement (ECGS) and Immobilon Western Chemiluminescent HRP (horseradish peroxidase) Substrate were purchased from Merck Millipore (Germany). Growth factor reduced (GFR) basement membrane matrix was purchased from Corning (Bedford, MA, USA). Crystal violet, heparin sodium salt, phosphatase inhibitors (PhosSTOP), and doxorubicin (Dox) were purchased from Sigma-Aldrich (Germany). Tris-buffered saline, RIPA buffer, bovine serum albumin (BSA), phosphate-buffered saline (PBS), and a prestained protein ladder were purchased from Bioman Scientific (Taiwan). A protease inhibitor cocktail was purchased from Bioshop (Canada). Cell Counting Kit (CCK)-8 was purchased from Dojindo Laboratories (Japan). Lipo-Dox (liposomal-encapsulated doxorubicin) was purchased from TTY Biopharm (Taiwan).

### 2.2 Synthesis of Mesoporous Silica NPs (MSNs)

Both 25- and 50 nm MSNs were synthesized by controlling the concentrations of TEOS and NH_4_OH according to our previous report.^30^ For 25 nm MSNs, 0.29 g of CTAB was dissolved in 150 mL of 0.128 M NH_4_OH, stirred for 15 min at 60 °C, and then 2 mL of 0.88 M TEOS in ethanol was added in a dropwise manner. For RITC conjugation, 8 mg of RITC dissolved in 5 mL of 99.5% ethanol was mixed with 10 μL of APTMS and stirred overnight in the dark. Afterward, 2.5 mL of APTMS-conjugated RITC was added before adding 2 mL of 0.88 M TEOS. The concentrations and temperature were changed (0.17 M NH_4_OH, 50 °C, 0.9 M TEOS) for 50 nm MSN synthesis. The reaction was stirred vigorously for 1 h. The surface was modified afterward by adding PEG-silane or PEG-silane and TA-silane to the solution and stirring for 30 min, and these NPs were respectively named MSN-PEG 25, MSN-PEG 50, MSN-PEG/TA 25, and MSN-PEG/TA 50. The solution was aged until the initial volume was reduced to 1/3 and then heated at 70 and 90 °C for 1 day each under hydrothermal conditions. Finally, HCl was added to remove surfactants (due to ion exchange) at 60 °C. After three ethanol-washing/centrifugation cycles, NPs were collected and stored in 99.5% ethanol.

### 2.3 Synthesis of Solid Silica NPs (SSNs)

SSNs (50 nm in diameter) were synthesized as described by the Stöber method. Briefly, 5.4 mL of H_2_O and 0.35 g of NH_4_OH (28–30 wt %) were added to 99.5% ethanol with a total volume of 25 mL and stirred for 10 min at 25 °C. Next, 25 mL of diluted TEOS solution (3.126 mL of TEOS in 22 mL of ethanol) was added rapidly and stirred for 1 h at 25 °C. Then 1 mL of silane-PEG was added to the solution in a dropwise manner under continued stirring for 1 h at 25 °C. Finally, the SSNs were washed with ethanol, centrifuged, and resuspended in 99.5% ethanol for later usage. These NPs were named SSN-PEG 50.

### 2.4 Characterization of MSNs

The morphology and mesoporous channels of MSNs and SSNs were imaged by transmission electron microscopy (TEM, Hitachi H-7100, Japan) operated at 75 keV. Micrograph-based NP size distributions were determined using Sigma Scan Pro 5.0 software (Ashburn, VA, USA). Dynamic light scattering (DLS, Zetasizer Nano ZS90, Malvern Instruments, UK) was used to determine the NP size of samples in deionized (DI) water and PBS (*n* = 3). N_2_ adsorption–desorption isotherms were measured at −196 °C (in liquid N_2_) using a Micromeritics ASAP 2020 instrument for surface area and porosity analyses, and Brunauer–Emmett–Teller (BET) and Barrett–Joyner–Halenda (BJH) methods were used for the calculation. The concentration of NPs was determined using a Nanoparticle Tracking Analysis (NTA, Malvern NanoSight NS300).

### 2.5 Preparation of Doxorubicin (Dox)-Loaded MSN-PEG/TA 25

MSN-PEG/TA 25 (20 mg) was dissolved in 2.8 mL of 0.1 M NaHCO_3_ (pH 9.95) for 30 min and then centrifuge-washed twice with double-distilled water (ddH_2_O). MSN-PEG/TA was mixed with 0.72 mg of Dox in 3 mL of ddH_2_O under continuous stirring for 1 h in the dark at 4 °C. After centrifugation and washing with ddH_2_O three times to remove free Dox, a product called Dox@MSN-PEG/TA 25 was obtained. The supernatant was collected, and the amount of free Dox was quantified against a standard calibration curve using a spectrophotometer (λ_ex_ = 480 nm, λ_em_ 560 nm). The loading amount and loading efficiency of Dox were calculated according to the following equations:
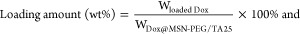

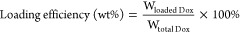


### 2.6 Cell Culture

4T1 cells (mouse mammary gland cancer cells) and OVCAR8 cells (an ovarian cancer cell line) were cultured in RPMI 1640 medium supplemented with 10% (v/v) FBS and 1% (v/v) penicillin-streptomycin. Human umbilical vein endothelial cells (HUVECs) were plated on 1% gelatin-coated tissue culture plates and maintained in 199 medium containing 20% (v/v) FBS, 30 μg/mL of ECGS, 25 U/mL heparin, and 1% (v/v) penicillin-streptomycin. All cells were purchased from American Type Culture Collection (Manassas, VA, USA) and incubated in a humidified atmosphere with 5% CO_2_ at 37 °C.

### 2.7 Cytotoxicity Assay

The cytotoxicity of NPs was determined by a CCK-8 assay. 4T1 cells were seeded in 96-well plates at a density of 10^4^ cells/well for 24 h. Different concentrations of NPs (100, 200, 500, and 1000 μg/mL) were incubated with cells for 24 h. Cells were washed twice with culture medium and incubated with CCK-8 reagent for 30 min at 37 °C, and the absorbance at 450 nm was measured with a microplate reader (model 680, Bio-Rad, USA) (*n* = 3).

### 2.8 Wound-Healing Assay

A wound-healing assay was performed using ibidi Culture-Inserts 2 Well (Germany). First, 4T1 cells (2 × 10^4^ cells/well) were seeded into both compartments of a Culture-Inserts 2 Well in 24-well plates for 24 h. After that, the Culture-Inserts were removed using tweezers to form a uniform wound. Cells were washed twice with PBS to remove detached cells and then cultured in RPMI medium with 1% FBS. After treatment with 200 μg/mL of MSNs or SSNs, images were acquired at 0, 16, and 24 h with a microscope (IX-71, Olympus), and the wound-healing ratio was quantitatively analyzed using ImageJ software (*n* = 3). The change in the wound area was calculated using the following equation:
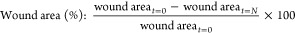


### 2.9 Cell Migration by a Boyden Chamber Assay

Prior to the Boyden chamber experiment, 4T1 cells were seeded in six-well plates (2 × 10^5^ cells/well) for 24 h and then treated with 200 μg/mL of MSNs or SSNs for another 24 h. After that, 4T1 cells were harvested in RPMI medium with 1% FBS and placed in the upper Boyden chamber (7 × 10^4^ cells/well). Complete RPMI medium with 10% FBS was added to the lower chamber as a chemoattractant. After 24 h of incubation at 37 °C, nonmigrated 4T1 cells on the upper side of the chamber were removed with a cotton swab. Optical images of 4T1 cell migration were taken after samples in the lower chamber were fixed in a 4% paraformaldehyde (PFA) solution and stained with 0.5% crystal violet. The percentage of the area covered by cell migration was analyzed colorimetrically by ImageJ software (*n* = 3).

### 2.10 Tube-Formation Assay

A tube-formation assay was performed by seeding HUVECs on Matrigel (50 μL/well) into a 96-well plate at 37 °C for 30 min to allow the Matrigel matrix to gel. A HUVEC density of 10^4^ cells/well was mixed with MSNs or SSNs (200 μg/mL) and then seeded into the Matrigel-precoated well. After incubation at 37 °C, tubules of HUVECs were imaged at 6 and 24 h with a microscope (IX-71, Olympus), and the number of junction points was quantified by ImageJ software (*n* = 3).

### 2.11 Real-Time Cell Analysis (RTCA)

An RTCA assay of cell migration was performed on an xCELLigence DP system according to the instrument’s manual (Roach Diagnostics, Germany). 4T1 cells (2 × 10^5^ cells/well) were seeded in six-well plates for 24 h and treated with 200 μg/mL of MSN-PEG/TA 25 for another 24 h. Then, cells were harvested in RPMI medium with 1% FBS, and 6 × 10^4^ cells/well were seeded in the upper chamber of cell invasion/migration (CIM)-plate 16. Complete RPMI medium with 10% FBS was used as a chemical attractant in the lower chamber. Cell index values were measured every 15 min for up to 28 h using RTCA software.

### 2.12 Three-Dimensional (3D) Live-Cell Holographic Imaging

HoloMonitor M4 holographic cytometry (Phase Holographic Imaging, Lund, Sweden) was housed in standard cell culture incubator conditions. Briefly, 4T1 cells were seeded at low confluency (10^5^ cells/dish) onto 35 mm culture dishes in RPMI medium overnight and then coincubated with 200 μg/mL of MSN-PEG/TA 25 for another 24 h. Next, dishes were directly placed onto a HoloMonitor M4 holographic microscope, and time-lapse phase images were recorded at intervals of 15 min over 24 h. For individual cell tracking, the center of mass was determined, and the cell motility at each time point was further analyzed by HStudioM4 software (*n* = 5).

### 2.13 Western Blot Analysis

4T1 cells treated with MSNs (200 μg/mL) for 24 h were washed twice with PBS and then harvested using cell scrapers. Cells were dispersed in 200 μL RIPA buffer (containing protease and phosphatase inhibitors) and kept on ice for 2 h for cell lysis. The supernatant of the cell lysate was collected after centrifugation, and the protein concentration was determined using a bicinchoninic acid (BCA) assay. Later, 20 μg of protein samples were mixed with loading dye, boiled for 10 min, and subjected to 10% sodium dodecyl sulfate polyacrylamide gel electrophoresis (SDS-PAGE). After electrophoresis for protein separation, proteins were electrophoretically transferred to a polyvinylidene difluoride (PVDF) membrane and blocked in 5% (w/v) BSA in TBST buffer [1× Tris-buffered saline, 0.1% Tween 20] for 1 h. The PVDF membrane was washed with TBST and incubated with different primary antibodies (Cell Signaling Technology, USA: e-cadherin, smad2, phosphorylated (p)-FAK, FAK, p-ERK, ERK, p-MAPK kinase (MEK), p-paxillin, and paxillin; Santa Cruz Biotechnology, USA: GAPDH, α-tubulin) overnight at 4 °C. After washing with TBST buffer three times, the PVDF membrane was incubated with a secondary immunoglobulin G (IgG) antibody (Santa Cruz Biotechnology) for 2 h at room temperature. Finally, immunoreactive bands were visualized with an enhanced chemiluminescence substrate kit (Amersham Pharmacia Biotech, GE Healthcare UK, Bucks, UK) according to the manufacturer’s protocol.

### 2.14 Real-Time Quantitative Polymerase Chain Reaction (qPCR)

4T1 cells were treated with MSNs (200 μg/mL) for 24 h and washed twice with ice-cold PBS. Cells were harvested and lysed in 1 mL of Azol RNA Isolation Reagent (Arrowtech, Taiwan). After RNA extraction, reverse transcription was performed using a cDNA Reverse Transcription Kit (Applied Biosystems) to synthesize complementary (c)DNA according to the manufacturer’s protocol. Expressions of specific genes were determined on a StepOne and StepOnePlus Real-Time PCR System with a SYBR Green FAST qPCR Master Mix Kit (KAPA Biosystems, USA). PCR conditions were heating at 95 °C for 3 min, followed by 40 cycles of 95 °C for 3 s and 60 °C for 30 s (*n* = 3).

### 2.15 Immunofluorescence Assay and Quantification

4T1 cells were treated with MSNs (200 μg/mL) for 24 h, washed with PBS twice, fixed with 4% PFA for 10 min, and then permeabilized with 0.1% Triton X-100 for 10 min. Cells were blocked with 3% BSA for 1 h and incubated with primary antibodies against paxillin (Cell Signaling Technology), F-actin (Alexa Fluor 488-labeled phalloidin, Thermo Fisher, USA), and α-tubulin (Cell Signaling Technology) overnight at 4 °C. Cells were extensively washed four times in TBST and further stained with Alexa Fluor-tagged secondary antibodies (paxillin, Alexa Fluor 488, shown in green; α-tubulin, Alexa Fluor 568, shown in red) for 1 h at room temperature. Nuclei were stained with DAPI (BioShop, Canada). Finally, cells were imaged using a fluorescence microscope (IX-71, Olympus), and quantified data corresponding to the number of FAs per cell were calculated (*n* = 3).

### 2.16 Animals

Female BALB/c mice (9 weeks old) were purchased from the BioLASCO Experimental Animal Center (BioLASCO, Taipei, Taiwan) and housed under specific pathogen-free (SPF) conditions. All experiments were performed according to the guidelines of the Laboratory Animal Center of Taipei Medical University.

### 2.17 In Vivo Multi-Photon Imaging of MSNs

4T1 xenograft BALB/c mice were intravenously injected with RITC-conjugated MSN-PEG/TA 25 at a dose of 200 mg/kg. Multiphoton laser scanning microscopy (LSM, Olympus FVMPE-RS), equipped with an IR laser with a tunable excitation wavelength ranging from 700 to 1080 nm, was used to capture images of the blood vessels in the mouse earlobe. Time-lapse images of the circulation of NPs were captured over a period of 10 to 120 min. Additionally, 4 and 24 h postinjection in mice, 60 μL of 2.5% (w/v) fluorescein isothiocyanate dextran (FITC-dextran, Mw: 70 kDa) dissolved in sterile saline was intravenously injected through the tail vein for blood vessel labeling. The images were observed using LSM.

### 2.18 *In Vivo* Antimetastatic and Antitumor Activities

For the metastasis assay, mice were subcutaneously implanted with 1.5 × 10^6^ luciferase-4T1 cells and then randomly assigned to different groups receiving MSNs (*n* = 6). First, MSN-PEG/TA 25 was intravenously (IV; 200 mg/kg) or intratumorally (IT; 20 mg/kg; high concentration: 200 mg/kg) injected into tumor-bearing mice on days 12, 15, and 18. The tumor size and body weight were measured during the experimental period at indicated times. The tumor volume was calculated using the formula:
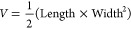
At days 21, 28, 32, and 35 after tumor implantation, in vivo imaging system (IVIS) images were taken to track tumor metastasis of mice. At the end point of the experiment, mice were sacrificed, and primary tumors were surgically removed and imaged. Then, the lungs of mice were injected with India Ink (15% India ink in 10% buffered formalin) via the trachea for quantification and imaging of the metastasis point of the lungs.^31−33^ Finally, the lungs were excised and fixed with 4% PFA for a histological analysis (hematoxylin and eosin (H&E) staining and immunochemical staining) with a primary Ki67 antibody (LifeSpan, Seattle, WA, USA). An Alexa Fluor-488 fluorescein-labeled secondary antibody (Invitrogen, USA) was administered, and nuclear counterstaining with DAPI (BioShop) was performed. Fluorescent images were obtained from an inverted fluorescence microscope (Olympus IX71).

### 2.19 Improvement in Overall Survival

Mice (*n* = 6)were implanted with 1.5 × 10^6^ luciferase-4T1 cells. On days 12, 15, and 18 postimplantation, for synergistic therapy with Lipo-Dox, mice received MSN-PEG/TA 25 by an IV (200 mg/kg) or IT (20 mg/kg) injection and combined with Lipo-Dox by an IV (4 mg/kg) injection. For Dox treatment, mice received free Dox (4 mg/kg) and Dox@MSN-PEG/TA (equivalent to 4 mg Dox/kg) by an IV injection. IVIS imaging, tumor volume, and body weight measurements were performed at the indicated times, and survival rates were monitored until the death of the last mouse. The median survival time (MST) was analyzed using the Kaplan–Meier method with Prism 9.4 software (GraphPad, US), defined as the time point at which 50% of subjects survive. The percentage increase in life span (%ILS) was determined using the following formula:
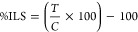
where *T* and *C* are the mean survival days of the treated and control groups of mice, respectively.

### 2.20 Immunofluorescence Staining of Tumors

Following the same treatment of MSN-PEG/TA 25 (20 mg/kg) three times by an IT injection in 4T1 xenograft mice, excised tumor tissues were fixed in 10% formalin, followed by paraffin embedding and sectioning. After washing, slices were incubated with p-paxillin, p-ERK, and p-FAK (Cell Signaling Technology) antibodies overnight at 4 °C. Slices were extensively washed and incubated with an Alexa Fluor-488 fluorescein-labeled secondary antibody (Invitrogen) at room temperature for 2 h, and nuclei were counterstained with DAPI (BioShop) for 10 min. Finally, fluorescent images were obtained with an inverted fluorescence microscope (Olympus IX71).

### 2.21 Biodistribution and Tumor Targeting of MSNs

4T1 xenograft mice were treated with RITC-conjugated MSN-PEG/TA 25 (200 mg/kg) by an IV injection. At 24 h postinjection, mice were euthanized, and the tumor and major organs, including the heart, liver, spleen, lungs, and kidneys, were excised. Additionally, blood and urine were collected from each sacrificed mouse. Images of MSN biodistributions were obtained with IVIS, and quantification of RITC fluorescence intensity was analyzed. The RITC signal was divided by each organ’s weight and then plotted as the ratio of each organ to the liver (organ/liver) (*n* = 3).

### 2.22 Anti-Angiogenesis in the Chick Chorioallantoic Membrane (CAM)

A CAM assay was used to study the effect of *in vivo* angiogenic activity by MSN-PEG/TA 25. Fertilized eggs were preincubated at 37 °C and 60% humidity for 10 days, and a small window of approximately 1 cm in diameter was created using a Dremel 3000 tool to cut the eggshell. A Teflon O-ring was placed onto the artery of the CAM membrane and subsequently seeded with 4T1 cells (3.5 × 10^6^ cells in 20 μL PBS). After that, the window was covered with Tegadern film (3M, USA), and the eggs were placed back into the incubator for solid tumor growth. The Teflon O-ring was removed 2 days after implantation (embryonic day 12). Dox (0.06 mg/egg) and MSN-PEG/TA 25 (1 and 1.5 mg/egg) were injected into the vein of the chorioallantoic membrane from the broadside of the egg through the opening pore with a syringe. Vascular plexus images on the CAM were photographed using an optical microscope on embryonic days 14 and 16. Blood vessel densities were calculated by NIH ImageJ software with the “angiogenesis analyzer” plug-in (*n* = 5).

### 2.23 Statistical Analysis

All data were analyzed using GraphPad Prism 9.0 software. All experiments were conducted in triplicate, and results are expressed as the mean ± standard deviation (SD). Statistical analysis in a two-group comparison was performed by a Student’s *t*-test. Statistical differences were shown as significant * *p* < 0.05, very significant ** *p* < 0.01, and highly significant *** *p* < 0.001.

## 3 Results and Discussion

### 3.1 Functionalized PEGylated Mesoporous Silica Nanoparticles Retard Cancer Metastasis

**Figure 1:**
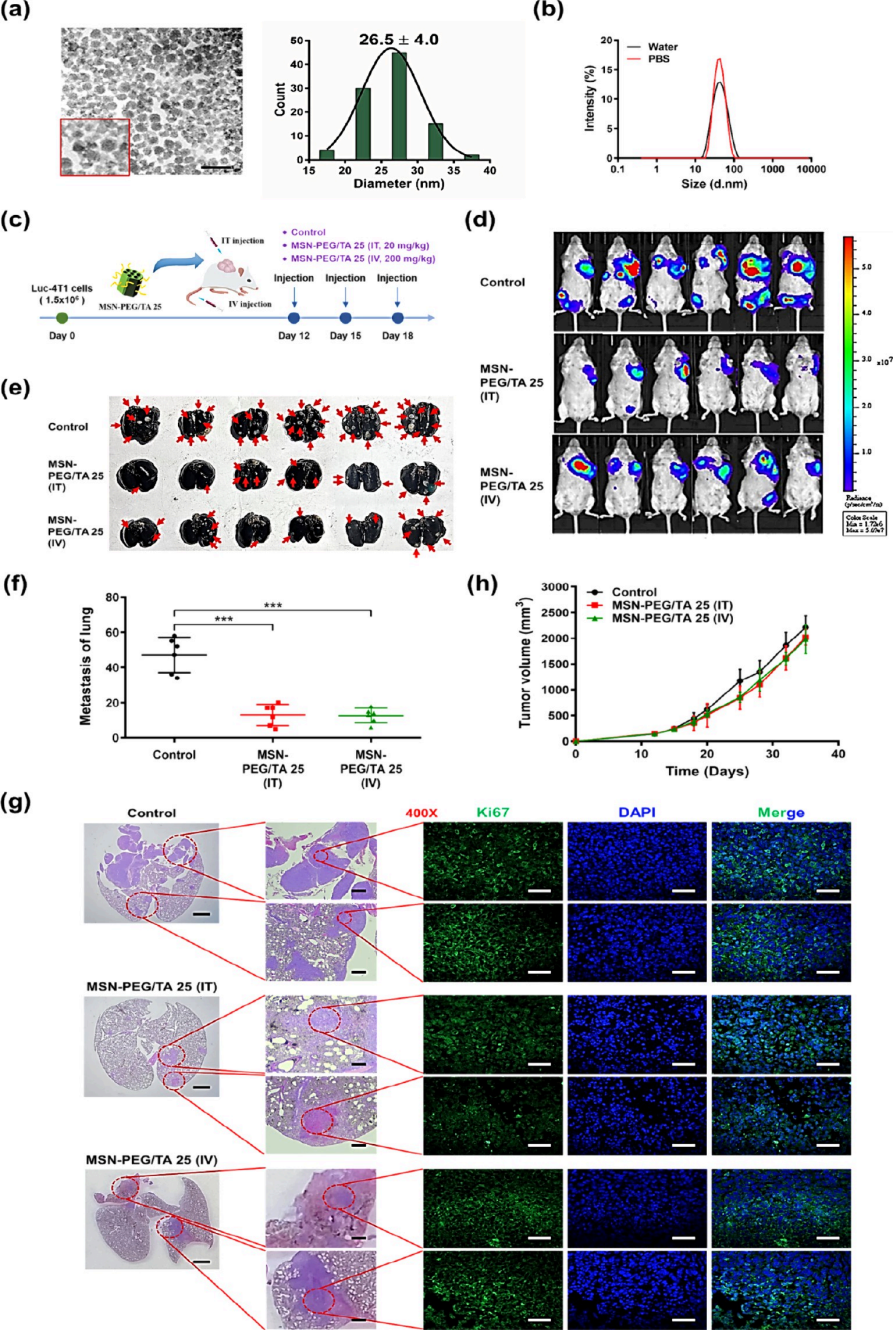
Antimetastatic activity of MSN-PEG/TA 25 in mice with 4T1 xenografts. (a) TEM micrographs illustrating the morphology of synthesized MSN-PEG/TA 25, accompanied by a particle size distribution histogram calculated using ImageJ software (scale bar: 100 nm). (b) The size distribution of MSN-PEG/TA 25 was measured via dynamic light scattering (DLS) in both deionized water and PBS (*n* = 3). (c) Experimental scheme. Mice were implanted with 1.5 × 10^6^ Luc-4T1 cells and then intratumorally (20 mg/kg) and intravenously (200 mg/kg) injected with MSN-PEG/TA 25 three times. At the end point (day 35), (d) IVIS images of a tumor and (e) an India ink-stained lung (front) were taken. Red arrows point to white metastatic nodules in the lung (*n* = 6). (f) Numbers of lung nodules with metastases (*** *p* < 0.001, *n* = 6). (g) H&E-stained sections of the metastatic lung were photographed (scale bar: 500 μm). Fluorescence images of sections were stained with *K*_i_ 67 (shown in green) for metastatic nodules in the lung and DAPI (shown in blue) for nuclei (scale bar: 50 μm). (h) Tumor volumes at different time points of treatments (*n* = 6).

According to our previous study, 25 nm diameter PEGylated mesoporous silica nanoparticles (MSNs) with quaternary amine modification of TA molecular (denoted as MSN-PEG/TA) were synthesized.^29,34^ The TEM images and particle size distribution histograms for MSN-PEG/TA 25 are depicted in Figure 1a, revealing a well-defined porous structure. The intensity-based, single-peak data of hydrodynamic size distributions indicated the good dispersion of MSN-PEG/TA 25 in both deionized water and PBS (Figure 1b).

To evaluate the effectiveness of in vivo MSNs antimetastasis treatment, we utilized a xenograft mouse model, replicating breast cancer with lung metastasis. The timeline for MSN treatment is depicted in Figure 1c. We implanted mice with luciferase (Luc)-labeled 4T1 breast cancer cells, which were grouped into control, IT, and IV injections of MSN-PEG/TA 25 (IT: 20 mg/kg; IV: 200 mg/kg). Bioluminescence images of the three groups of mice were recorded on days 21, 28, 32, and 35 (Figure S1a). The control group exhibited the highest bioluminescence signals and spread to other body parts, indicating severe metastasis. By day 35, metastatic activity of MSN-PEG/TA-treated mice, both IT and IV, was greatly reduced compared to the control group (Figure 1d).

Since the lungs are the major metastatic organ for 4T1 breast cancer xenografts,^35,36^ we quantified metastatic nodules in the three groups at the end of the experiment by staining them with Indian ink. The stained lungs in Figure 1e and Figure S1b show significantly more white metastatic nodules (indicated by red arrows) in the control group than in those treated with MSN-PEG/TA 25 NPs (both IV and IT injections). Quantitatively, the control group exhibited an average of 47% nodules per lung, while MSN-PEG/TA treatments reduced the average to only about 13% for both IV and IT injections, translating to a 3.6-fold decrease in lung metastasis by MSN-PEG/TA 25 treatment (Figure 1f). We further examined metastatic lesions in the lungs by H&E staining using Ki67, a tumor proliferation marker, confirming that the nodules were tumor tissues (Figure 1g). However, the volumes and images of the primary tumors (Figure 1h and Figure S1c) in all three groups increased over time, indicating no therapeutic effect of MSN-PEG/TA treatment on the primary tumor. The absence of primary tumor growth inhibition with drug-free MSN-PEG/TA 25 is expected, as it lacks chemotherapeutic agents. To assess the biosafety of MSNs, we monitored the body weight of mice in all three groups during treatment. No significant differences were observed among the groups, as shown in Figure S1d, suggesting no significant systemic toxicity. Remarkably, the in vivo results agreed with the in vitro findings, demonstrating MSN-PEG/TA 25′s potential in restraining tumor metastasis in xenograft mice. Notably, a higher concentration of MSN-PEG/TA 25 (200 mg/kg) by IT injection did inhibit metastasis (Figure S2). However, the results were similar to those observed at a lower concentration (20 mg/kg), suggesting an upper limit on efficacy. These findings are crucial, demonstrating MSN-PEG/TA 25′s effectiveness in restricting metastasis. Given that MSN-PEG/TA 25 can serve as a nanocarrier for anticancer drugs, we anticipate that this antimetastatic NP could significantly improve cancer treatment outcomes when loaded with drugs used in chemotherapy or targeted therapy.

### 3.2 MSN-PEG/TA 25 Targets Tumors Through an Enhanced Permeation and Retention (EPR) Effect and Affect Cell Behavior

**Figure 2:**
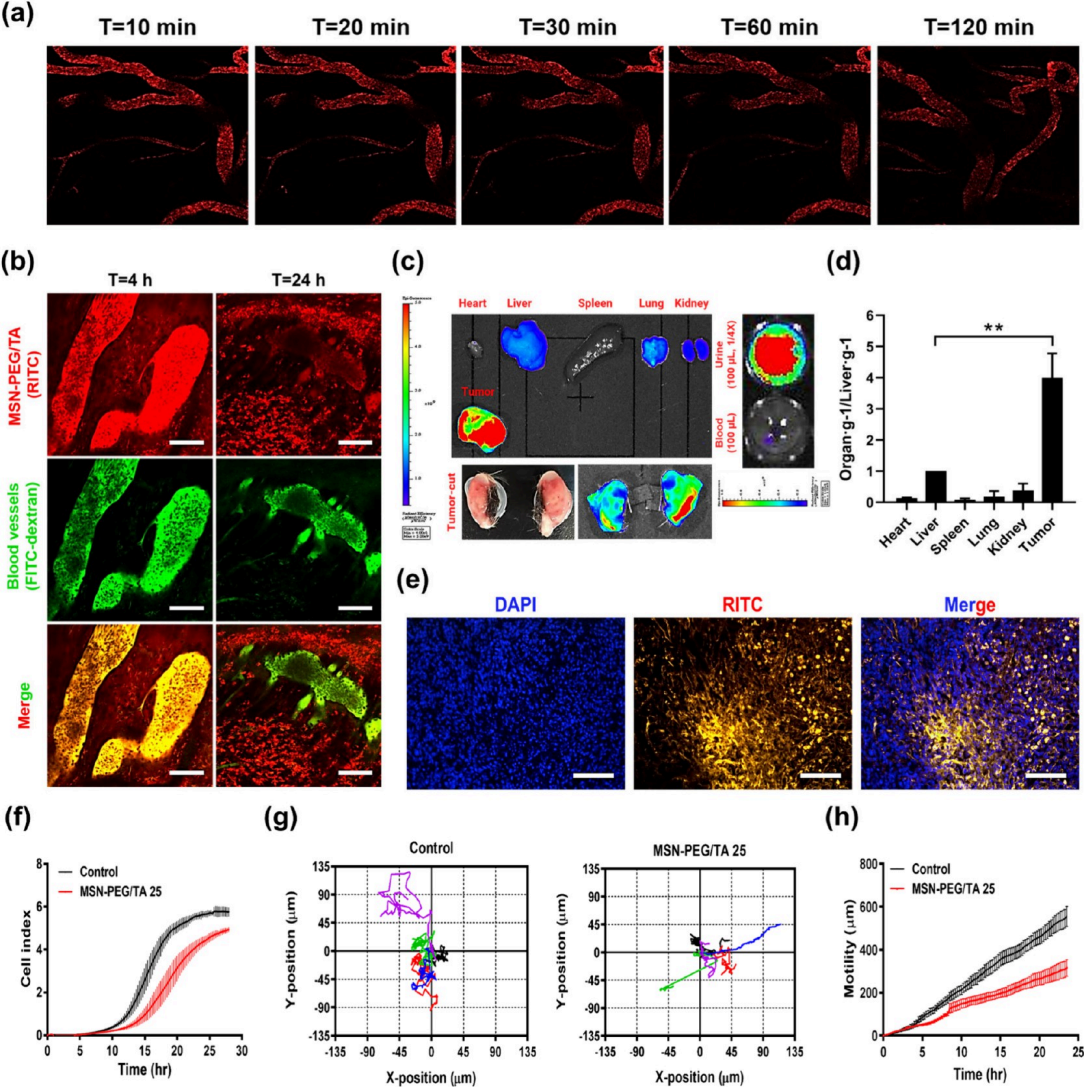
Passive tumor targeting in 4T1 xenograft mice and dynamics monitoring of cell behavior in 4T1 cells after treatment with MSN-PEG/TA 25. Mice were implanted with 1.5 × 10^6^ 4T1 cells and then intravenously injected with RITC-conjugated MSN-PEG/TA 25 (200 mg/kg body weight). (a) Multiphoton laser scanning microscopy imaged the circulation of NPs inside the blood vessels in the ears of live mice within 10 to 120 min. (b) Visualization of the enhanced permeability and retention (EPR) effect. Blood vessels were stained with fluorescein isothiocyanate dextran (FITC-dextran) dye (green) via IV injection, and detailed enlarged images of RITC-conjugated MSN-PEG/TA 25 (red) localization were observed using multiphoton laser scanning microscopy 4 and 24 h postinjection in mice. The overlap of orange signals demonstrated the colocalization of MSN-PEG/TA 25 (red) with the blood vessels (green). Scale bar: 100 μm. (c) Biodistribution of RITC-conjugated MSN-PEG/TA 25 was captured using an IVIS analysis after 24 h of treatment. (d) Quantification of the fluorescence intensity. The RITC signal was divided by the weight of each organ and plotted as the ratio of each organ to the liver (organ/liver). (**p < 0.01, *n* = 3). (e) Fluorescence microscopic images of excised tumors stained with DAPI (nuclei, blue). Yellow signals represent RITC-conjugated MSN-PEG/TA 25. Scale bar: 100 μm. Additionally, 4T1 cells were treated with and without 200 μg/mL of MSN-PEG/TA 25 for 24 h. (f) Real-time cell analysis (RTCA) assay indicating the cell migration index of 4T1 cells. Holographic microscopic analysis of (g) the directionality of motility plots and (h) the distance of motility over time. Each colored line represents the paths of a single-cell migration (*n* = 5).

The therapeutic efficacy of nanomedicine relies on the tumor targeting ability of NPs, a step that takes precedence over their antimetastatic ability. Previous studies have demonstrated that silica NPs with a 20 nm diameter outperform those with 50- and 200 nm diameters in terms of cellular uptake, prolonged blood circulation, and tumor targeting.^29,37^ To evaluate the efficacy of MSN-PEG/TA 25 as an effective tumor-targeting agent, we conducted an in vivo experiment by implanting 4T1 cells in mice. The mouse models were intravenously administered RITC-conjugated MSN-PEG/TA 25 at a dose of 200 mg/kg. Red signals of MSN-PEG/TA 25 were observed using multiphoton laser scanning microscopy (LSM) from 10 to 120 min postinjection, indicating good circulation (Figure 2a). To determine whether the MSNs had leaked from the blood vessels, we stained the blood vessels following the injection of fluorescein isothiocyanate dextran dye (FITC-dextran, Mw: 70 kDa) 4 and 24 h postinjection in mice. The orange signals from MSN-PEG/TA 25 (red, RITC) overlapped with the distribution of blood vessels (green, FITC-dextran), revealing that some of the NPs remained circulating inside the blood vessels (T = 4 h). Notably, MSN-PEG/TA 25 could be located inside cells near the blood vessels (T = 24 h), suggesting leakage from the blood vessels and uptake by tumor cells (Figure 2b and Figure SI: video 1).

Further, the biodistribution of MSN-PEG/TA 25 was examined 24 h postinjection using IVIS. Results are illustrated in Figure 2c, where RITC-conjugated MSN-PEG/TA 25 was found to accumulate at tumor sites via an enhanced permeation and retention (EPR) effect. The fluorescence signals in tumors relative to the liver were exceptionally high, at a ratio of 4.0. In contrast, organs like the heart, spleen, lungs, and kidneys showed ratios below 0.5, as depicted in Figure 2d, indicating the specific passive targeting capabilities of MSN-PEG/TA 25. Notably, the amount of MSN-PEG/TA 25 measured in urine was higher than in blood, suggesting possible kidney excretion. However, the exact renal clearance route remains unknown, and further studies are required to identify the clearance mechanism. To further confirm the tumor targeting of MSN-PEG/TA 25 via the EPR effect, we stained tissue sections with 4′,6-diamidino-2-phenylindole (DAPI, blue color) to visualize tumor cell nuclei (Figure 2e). The tumor region, identified by dense DAPI staining, displayed numerous RITC-NP signals (yellow). This significant presence of MSN-PEG/TA 25 in the tumor area strongly suggests that these NPs effectively target tumors through the EPR effect.

Knowing that MSN-PEG/TA 25 can target 4T1 breast tumors by EPR effect and suppress tumor metastasis, we further investigated the internalization of NPs and their influence on cell migration. Using real-time cell analysis (RTCA), we monitored the dynamic behavior of highly metastatic 4T1 breast cancer cells. As illustrated in Figure 2f, 4T1 cells exhibited rapid migration around 7 h postseeding in the control group. In contrast, MSN-PEG/TA 25-treated cells showed delayed migration. The cell indexes of MSN-PEG/TA 25-treated cells remained lower than those of the control group even after reaching a plateau, indicating that the mobility of 4T1 cells was hindered by MSN-PEG/TA 25 treatment. To monitor individual cell movement, real-time cell tracking with a phase holographic microscope (HoloMonitor M4) was used to capture time-lapse images (Figure SI: video 2). Results depicted in Figure 2g show that cells in the MSN-PEG/TA 25-treated group moved within a limited area compared to the control group. The motility of cells, shown in Figure 2h, also confirmed that the migratory activity of 4T1 cells was hindered by MSN-PEG/TA 25 treatment, making it a potential candidate for antitumor metastasis applications.

### 3.3 Synthesis and Characterization of Various MSNs for Antimetastasis Assessment

**Figure 3:**
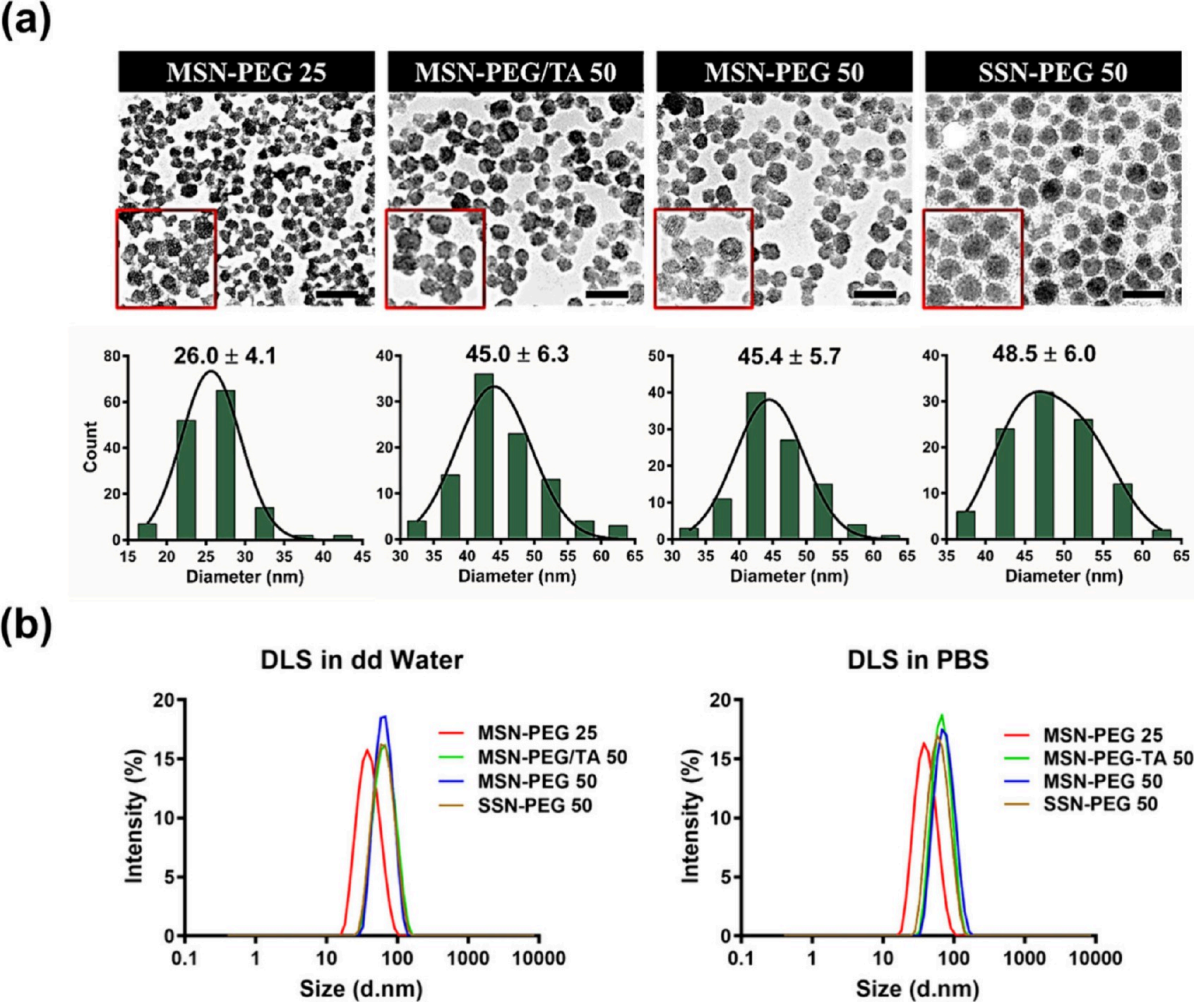
Characterization of various types of silica NPs. (a) TEM micrographs showing the morphology of synthesized mesoporous silica NPs (MSNs) and solid silica NPs (SSNs) with their particle size distribution histogram calculated using ImageJ software (scale bar: 100 nm). (b) Size distribution of NPs measured by dynamic light scattering (DLS) in double-distilled water and PBS (*n* = 3).

Inspired by findings that drug-free MSN-PEG/TA 25 might inhibit cancer metastasis, we aimed to explore the unique features of NPs, focusing on the impacts of particle sizes, surface alterations, and mesoporous structures on cancer metastasis. Accordingly, we prepared three additional types of MSNs with different sizes and surface modifications, following procedures described in the “Experimental section.″ These included MSNs of 25 and 50 nm with surface modifications using PEG-silane and TA-silane (denoted MSN-PEG/TA 50) and those solely with PEG-silane (MSN-PEG 25 and MSN-PEG 50). As a control, we synthesized 50 nm solid silica NPs (SSNs) with a PEG-silane surface, referred to as SSN-PEG 50. TEM images and particle size distribution histograms for the various MSNs and SSN-PEG 50 are shown in Figure 3a. Each type of MSN displayed a well-defined porous structure, in contrast to the solid structure of SSN-PEG 50. The intensity-based, single-peak data of hydrodynamic size distributions confirmed the stability of these NPs in deionized water and PBS (Figure 3b). Table 1 summarizes the TEM-based diameters and DLS-based hydrodynamic diameters of the 25- and 50 nm NPs, as well as their surface areas (SBET) and pore diameters (DBJH) obtained through nitrogen adsorption–desorption isotherms. The characteristic type IV isotherm with a hysteresis loop (Figure S3) confirmed the mesoporosity of the materials. Hydrodynamic diameters of the 25- and 50 nm NPs were approximately 40 and 60 nm, respectively, with no significant variance within MSNs of the same size.

**Table 1:** Physicochemical Characterization of Silica Nanoparticles^a^

	**D**_**h**_**in H**_**2**_**O/PBS**		**TEM size**		
**Sample**	Z-avg (d.nm)	PDI	Diameter (nm)	**S_BET_ (m^2^/g)**	**D_BJH_ (nm)**
MSN-PEG/TA 25	39.8/39.4	0.14/0.11	26.5 ± 4.0	354	1.58
MSN-PEG 25	37.3/37.8	0.11/0.13	26.0 ± 4.1	553	2.00
MSN-PEG/TA 50	61.5/64.7	0.09/0.06	45.0 ± 6.3	354	1.44
MSN-PEG 50	61.6/63.2	0.05/0.10	45.4 ± 5.7	732	2.00
SSN-PEG 50	59.2/59.3	0.11/0.11	48.5 ± 6.0	22	N/A

a Abbreviations: Dh, hydrodynamic size; Z-avg, intensity weighted harmonic mean size; PDI, polydispersity index; SBET, surface area; DBJH, pore diameter; N/A, not applicable.

Notably, 50 nm MSNs exhibited a greater surface area than 25 nm MSNs, attributed to their larger size, increased internal pore volume, and extensive internal surface area. Additionally, MSN-PEG/TA NPs displayed a reduced surface area and pore diameter, likely due to TA-silane grafting within the MSNs’ internal pores. Consequently, the surface area and pore diameter of MSN-PEG/TA NPs were significantly smaller than those of MSN-PEG. As evidenced by TEM, SSNs lacked a porous structure, resulting in a markedly reduced surface area of 22.4 m2/g, substantially lower than those of the MSNs. The thermogravimetric analysis (TGA) results, illustrated in Figure S4, indicated three distinct weight loss stages between 40 and 800 °C, further explained in Table S1. The initial stage (40–200 °C) involved loss of surface-bound water and residual organic solvents for all samples.^38,39^ The subsequent phase, spanning 200 to 500 °C, corresponded to the decomposition of functional groups on MSNs and SSNs. Weight reductions for various MSNs ranged from 25% to 29%, with SSN-PEG 50 showing a deviation of 17.80%. This discrepancy was likely due to the different surface areas between MSNs and SSNs, which influenced the quantity of functional groups on the NPs. NTA results (Figure S5) indicated estimated NP concentrations of 7.96 × 10^8^, 4.87 × 10^8^, 4.80 × 10^9^, 4.01 × 10^9^, and 1.37 × 10^9^ NPs/mL for MSN-PEG/TA 25, MSN-PEG 25, MSN-PEG/TA 50, MSN-PEG 50, and SSN-PEG 50, respectively. Cytotoxicity assessments of NPs at different concentrations (100, 200, 500, and 1000 μg/mL) revealed negligible cytotoxicity against 4T1 cells (Figure S6), suggesting that the antimetastatic actions observed in subsequent experiments were not related to NP-induced cytotoxicity.

### 3.4 MSNs Inhibit the Migration of 4T1 Cells and Tube Formation of HUVECs

**Figure 4:**
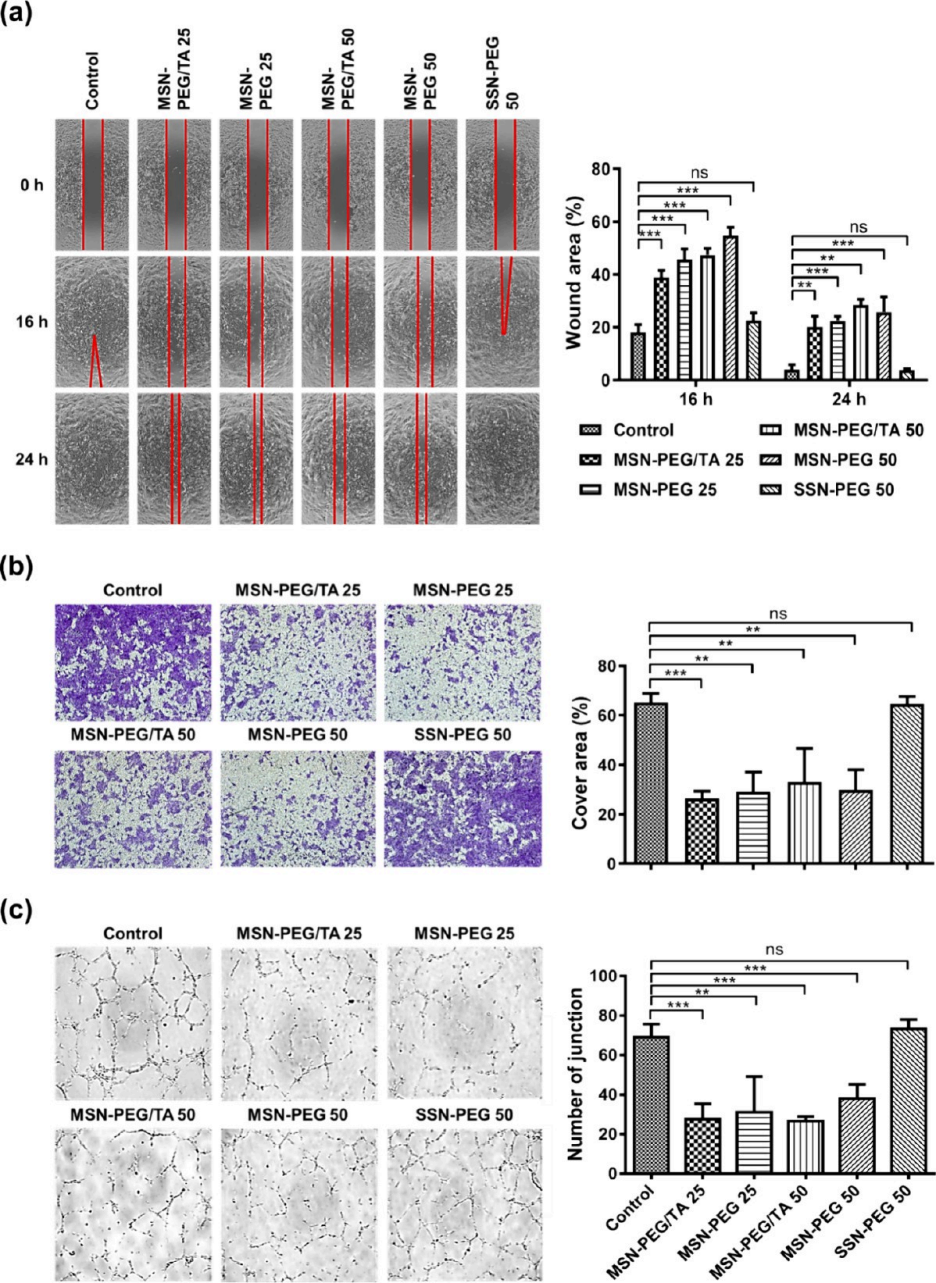
Cell migration and angiogenesis effects upon treatment with silica NPs. (a) Wound-healing assay. Uniform wounds of 4T1 cells were created using ibidi Culture-Inserts 2 Wells, and the cells were coincubated with various silica NPs (200 μg/mL) for 0, 16, and 24 h. Optical images of cell migration (left) and quantitative analysis of wound areas enumerated by ImageJ software (right). (b) Boyden chamber assay. After 24 h of treatment, migrated cells were fixed in a 4% PFA solution and then stained with 0.5% crystal violet. Optical images of migrated 4T1 cells on the lower chamber (left) and the percentage of the area covered by migrated cells (right). (c) Tube-formation assay. HUVECs were incubated with various silica NPs (200 μg/mL) for 24 h and then plated on 96-well Matrigel-coated plates. Optical images of the tube (right) and the number of tube junctions counted by ImageJ software. (**p < 0.01, ***p < 0.001, *n* = 3).

To evaluate the migration inhibition capability of MSNs, we utilized wound-healing assays with 4T1 cells. For comparison, we also tested the SSN-PEG 50. Results are shown in Figure 4a, where a notable retardation in wound-healing rates with MSN (200 μg/mL) treatment was observed. After 16 h, MSN-treated groups retained about 50% of the original wound area, in contrast to 20% in the control and SSN-PEG 50 (200 μg/mL)-treated groups. After 24 h, the wounds had almost completely closed in the control and SSN-PEG 50 groups, but about 20% remained in the MSN-treated groups. To further validate that MSNs could inhibit 4T1 cell penetration, a Boyden chamber assay was employed (Figure 4b). Results showed that all four types of MSNs (200 μg/mL) with different sizes and surface modifications could substantially inhibit 4T1 cell penetration, whereas SSN-PEG 50 (200 μg/mL) did not. All four types of MSNs hindered 4T1 cell migration regardless of the size or surface modification. Since differences between the MSNs and SSNs mainly involved surface structures, we speculated that the mesoporous structure of MSNs might be a key feature responsible for inhibiting cancer cell migration and penetration.

Knowing that MSNs can inhibit the migration of cancer cells, they may also influence the migration of endothelial cells, which is essential for tumor angiogenesis, and leads to tumor growth and metastasis. It was reported that MSNs can modulate endothelial cells’ angiogenic behavior through size control.^28^ We wondered if our MSNs could also hamper the angiogenic properties of endothelial cells. To assess the antiangiogenic effects of MSNs, a tube formation assay was used, where HUVECs were cultured on a Matrigel-coated plate, and the number of tube junctions was quantified. As illustrated in Figure 4c, all four types of MSNs (200 μg/mL) drastically impeded tube formation, unlike SSN-PEG 50 (200 μg/mL). These results suggest that MSNs are better at modulating cancer cell migration and endothelial tube formation than their solid-structured counterparts. Therefore, MSNs may be used for antimetastatic treatments.

### 3.5 Combining MSN-PEG/TA 25 with Lipo-Dox Enhances Survival in Mice with 4T1 Xenografts

**Figure 5:**
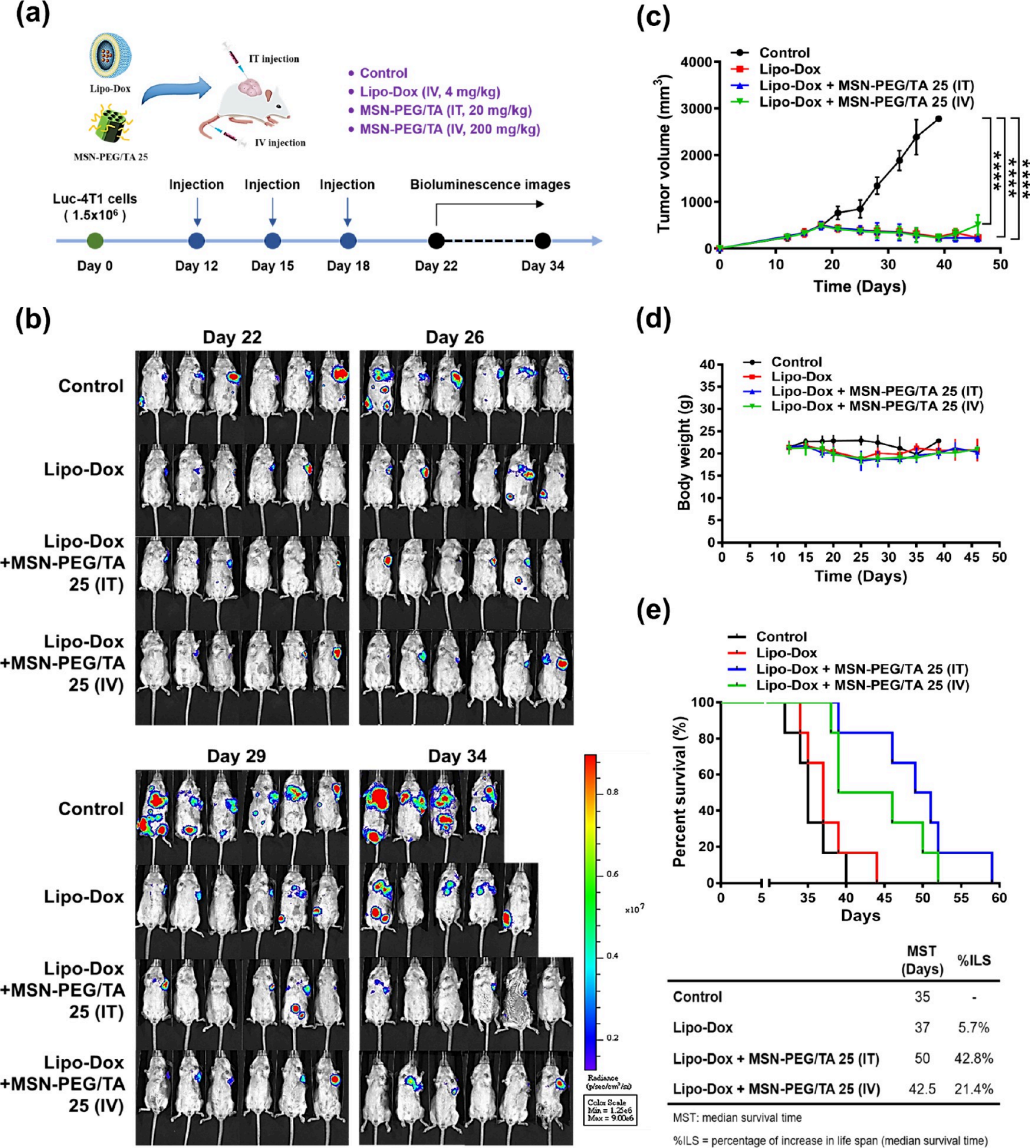
Synergistic effect of cotreatment of liposome-encapsulated doxorubicin (Lipo-Dox) and MSN-PEG/TA 25 in mice with 4T1 xenografts. (a) Schematic diagram of the experimental design: mice were implanted with 1.5 × 10^6^ Luc-4T1 cells and then intravenously injected with Lipo-Dox (4 mg/kg) in combination with MSN-PEG/TA 25 (200 mg/kg) intravenously or MSN-PEG/TA 25 (20 mg/kg) intratumorally three times at 3-day intervals. (b) IVIS image of tumor distribution within 22 to 34 days (*n* = 6). (c) Tumor volume, (d) body weight, and (e) Kaplan–Meier plots of overall survival, median survival time (MST), and percent increase in life span (% ILS) of mice (**** *p* < 0.0001, *n* = 6).

To evaluate the therapeutic applications of MSNs, we first investigated the synergistic effect of combining MSN-PEG/TA 25 with the existing chemotherapeutic drug, Dox, in mice with Luc-4T1 xenografts. Mice were IV treated with 4 mg/kg of Lipo-Dox (liposome-encapsulated Dox) and a combination of Lipo-Dox and MSN-PEG/TA 25 (200 mg/kg). Additionally, a group of mice was treated with an IV injection of Lipo-Dox (4 mg/kg) and an IT injection of MSN-PEG/TA 25 (20 mg/kg). The drugs were administrated three times in all three groups (Figure 5a). Bioluminescence images of the three drug-treated groups and a control group were recorded on days 22, 26, 29, and 34, as illustrated in Figure 5b. By day 26, cancer metastasis was evident in both the control and Lipo-Dox groups, with mouse deaths occurring by day 34. As expected, the control group (untreated) demonstrated a higher metastasis rate. In contrast, combined treatment with Lipo-Dox and MSN-PEG/TA 25, whether administered by IV or IT, resulted in a marked reduction of metastasis, indicating the synergistic effect of the combined treatment. Tumor volumes were reduced in both the Lipo-Dox only and combined treatment groups due to Lipo-Dox’s inherent cytotoxicity (Figure 5c). Body weight in all groups remained relatively stable (Figure 5d). Importantly, as depicted in Figure 5e, overall survival rates of mice treated with the combined therapy significantly improved compared to those receiving only Lipo-Dox. Although the combined treatment did not surpass Lipo-Dox monotherapy in terms of tumor size reduction or weight maintenance, the overall survival enhancement was remarkable. Additionally, survival analyses were conducted by measuring the median survival time (MST) and the percent increase in life span (% ILS), which are standard criteria in preclinical survival studies. The MST of control mice was 35 days. Administration of Lipo-Dox did not effectively increase survival (MST = 37 days, % ILS = 5.7%). However, the combination of Lipo-Dox with MSN-PEG/TA 25 (administered by IV or IT) significantly prolonged the MST to 42.5 and 50 days (% ILS = 21.4% and 42.8%, respectively). Our results demonstrated that the antimetastatic properties of MSN-PEG/TA 25 improved overall survival in mice with 4T1 xenografts.

### 3.6 Dox-Loaded MSN-PEG/TA 25 Suppresses Primary Tumors and Metastasis in Mice with 4T1 Xenografts

**Figure 6:**
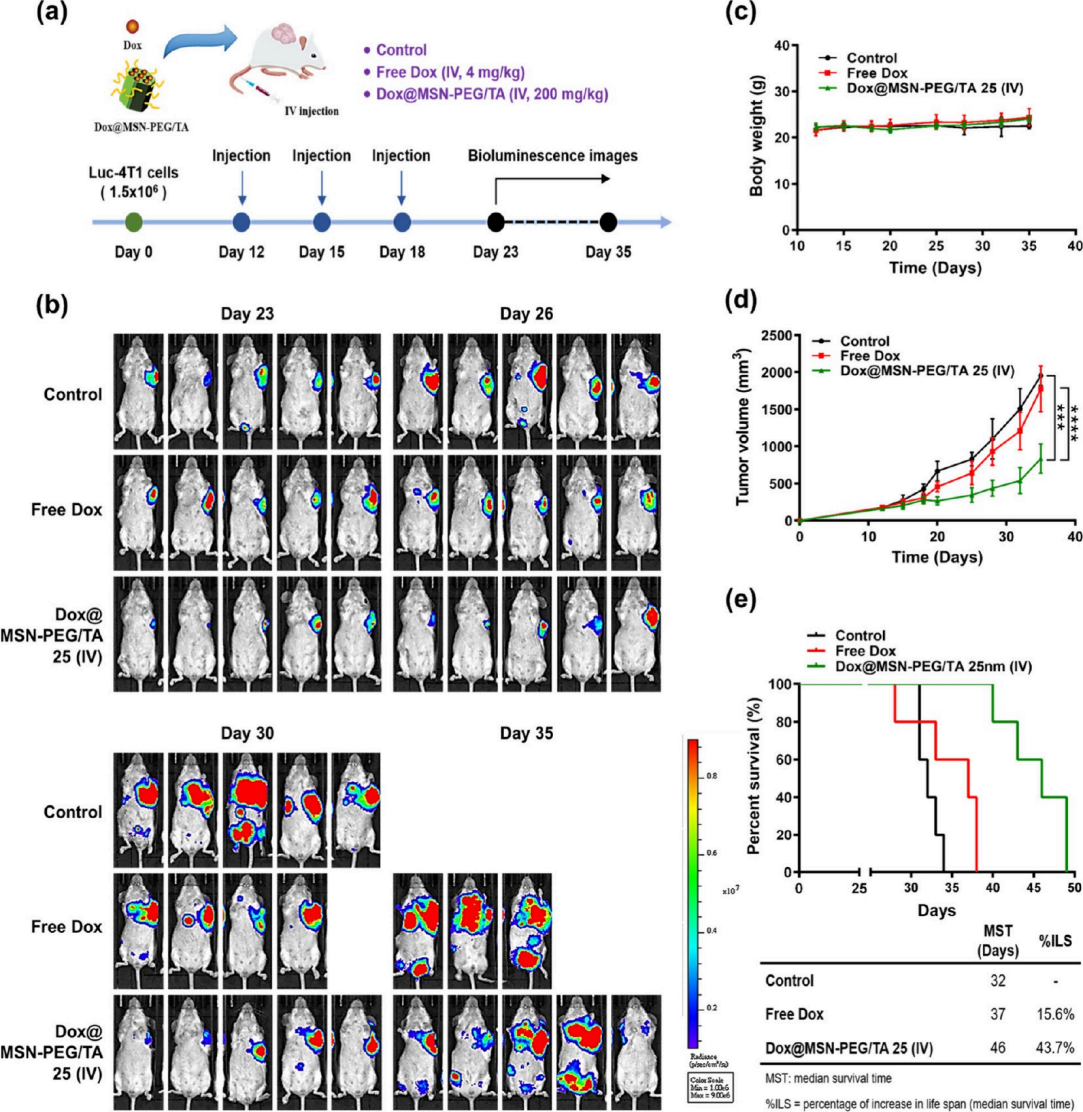
Antitumor activity of doxorubicin (Dox)-loaded MSN-PEG/TA 25 in mice with 4T1 xenografts. (a) Schematic diagram of the experimental design: Mice were implanted with 1.5 × 10^6^ Luc-4T1 cells and then intravenously injected with free Dox (4 mg/kg body weight) and Dox@MSN-PEG/TA 25 (200 mg/kg, equivalent to 4 mg of Dox) three times at 3-day intervals. (b) IVIS image of the tumor distribution within 23 to 35 days (*n* = 5). (c) body weight, (d) tumor volume, and (e) Kaplan–Meier plots of overall survival, median survival time (MST), and percent increase in life span (% ILS) of mice (*** *p* < 0.001, **** *p* < 0.0001, *n* = 5).

Given that combined treatment with Lipo-Dox and MSNs could improve the overall survival rate of mice with 4T1 xenografts, we further explored the efficacy of Dox-loaded MSNs, which are popular nanocarriers used in nanomedicine. Since the dosage used in the combined treatment was 200 mg/kg for MSN-PEG/TA 25 and 4 mg/kg for free Dox, we loaded Dox into MSNs to form Dox@MSN-PEG/TA 25 to achieve a similar concentration, which contained roughly 2 wt % of Dox with a 57% loading efficiency. Upon IV administration of either free Dox (4 mg/kg) or an equivalent Dox dosage within Dox@MSN-PEG/TA 25 (200 mg/kg) to mice with 4T1 xenografts, we observed distinct therapeutic results (Figure 6a). By day 30, tumor signals in the group treated with Dox@MSN-PEG/TA 25 were significantly smaller than those in the group treated with free Dox, as seen in bioluminescence images (Figure 6b). Body weight analysis (Figure 6c) indicated negligible toxicity in all groups. Meanwhile, Figure 6d illustrates that Dox@MSN-PEG/TA 25 significantly reduced tumor size, whereas the group treated with free Dox showed only slight tumor reduction, indicating partial inhibition of tumor growth by free Dox. We attributed the reduction in tumor size by Dox@MSN-PEG/TA 25 to the EPR effect, which causes NPs like MSNs to accumulate in tumors. The overall survival rates for the three groups are illustrated in Figure 6e. The efficacy of Dox@MSN-PEG/TA 25 treatment was significantly better compared to the free Dox and control groups. The control and free Dox groups showed mortalities between days 30 and 40, primarily due to extensive tumor growth and metastasis. Although free Dox did suppress primary tumors, its antimetastatic potential seemed limited. In contrast, Dox@MSN-PEG/TA 25, combining the antitumor power of Dox and the antimetastatic strength of MSN-PEG/TA 25, demonstrated an average 11-day survival advantage over the free Dox group. Survival analyses included measuring the median survival time (MST) and the percentage increase in life span (%ILS). The MST of control mice was 32 days. Administration of free Dox did not significantly increase survival (MST = 37 days, %ILS = 15.6%). Dox@MSN-PEG/TA 25 (administered intravenously) significantly prolonged the MST to 46 days (%ILS = 43.7%). Thus, the dual therapeutic potential of Dox@MSN-PEG/TA 25 is promising for cancer treatment.

### 3.7 Understanding the Mechanism Behind MSN-PEG/TA 25′s Impact on Cell Migration

**Figure 7:**
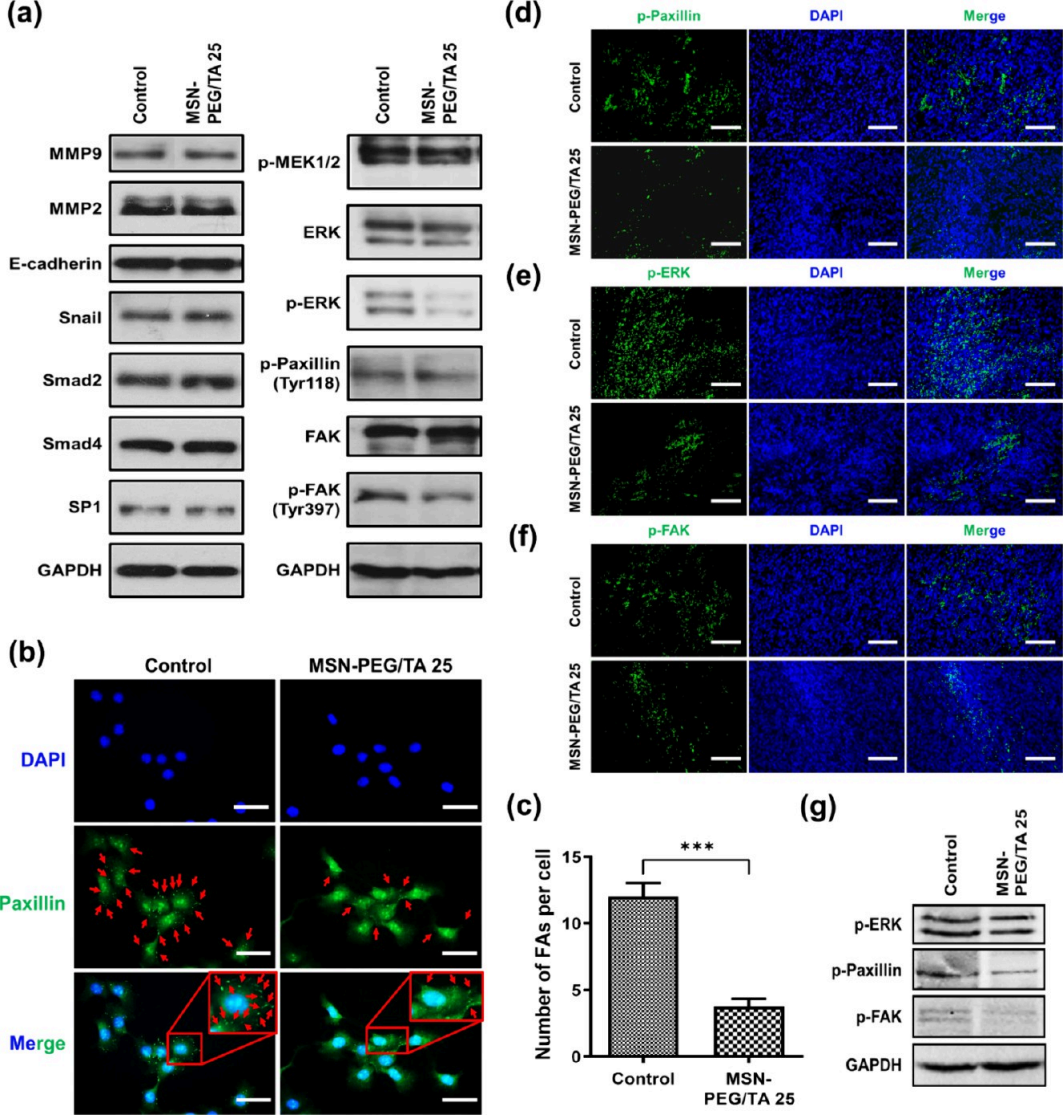
Mechanistic study of MSN-PEG/TA 25 on metastasis and focal adhesion turnover *in vitro* and *in vivo*. 4T1 cells were treated with 200 μg/mL of MSN-PEG/TA 25 for 24 h. (a) Western blot analysis of expression levels of various proteins related to metastasis and focal adhesion turnover. (b) The expression and localization of paxillin (green) and nuclei (blue) were imaged by an immunofluorescence assay. Scale bar: 50 μm. (c) The number of focal adhesions per cell. (*** *p* < 0.001, *n* = 3). Also, the expressions of focal adhesion turnover-related proteins in tumors were performed. Mice were implanted with 1.5 × 10^6^ of Luc-4T1 cells and then intratumorally injected with MSN-PEG/TA 25 (20 mg/kg) three times. Fluorescence microscopic images of excised tumors stained with (d) phosphorylated (p)-paxillin, (e) p-extracellular-regulated kinase (ERK), and (f) p-focal adhesion kinase (FAK) (green); nuclei (blue). Scale bar: 100 μm. (g) Western blot analysis of p-paxillin, p-ERK, and p-FAK. GAPDH was used as a loading control.

Our in vitro and in vivo results clearly demonstrate that MSN-PEG/TA 25 can inhibit metastasis. However, the molecular mechanisms behind this antimetastatic effect remain unclear. To explore these mechanisms, we first examined the mRNA expression levels of genes related to cancer metastasis. We assessed the mRNA expression in 4T1 cells treated with 200 μg/mL of MSN-PEG/TA 25 for 24 h using qPCR. Surprisingly, no significant differences were found in the expression levels of genes associated with the TGF-β/Smad signaling pathway (TGF-β1, SMAD4, and SMAD2), metastasis-related transcription factors (SP1) and genes (ITGβ1 and HPSE), extracellular matrix (ECM) degradation-related genes (TIMP3, MMP13, MMP10, and MMP3), or proinflammatory cytokines (IL1β and IL18) (Figure S7). These findings were supported by Western blot analyses (Figure 7a). Additionally, an analysis of protein expression levels revealed no differences in cancer metastatic processes, including the epithelial-mesenchymal transition (EMT) (E-cadherin and Snail) and ECM degradation (MMP) with or without MSN-PEG/TA 25 treatment. Our results suggested that the above pathways were probably not involved in the antimetastatic effect of MSNs. Therefore, we suspected that MSNs might directly affect cell motility.

Focal adhesion kinase (FAK) is a crucial regulator of cellular migration and adhesion, promoting cancer progression.^40^ It was found that FAK ablation in carcinoma led to impaired metastasis in an FAK-knockout mouse model.^41^ Several studies have reported that post-translational phosphorylation of proteins such as MEK1/2, ERK, paxillin, and FAK is essential for the turnover of FAs during cell locomotion.^42−46^ Therefore, we examined the expressions of FA-related proteins. We noted reductions in phosphorylated levels of ERK, paxillin, and FAK in MSN-PEG/TA 25-treated cells compared to the controls (Figure 7a), suggesting a possible role for MSNs in influencing FA turnover.

Since the dynamic remodeling of FAs is a critical process in regulating cell migration, we investigated paxillin, an essential protein in FAs that coordinates the assembly and disassembly of FAs, using immunostaining. As shown in Figure 7b, paxillin (green, red arrow) in 4T1 cells, with nuclei labeled by DAPI (blue), decreased when cells were treated with MSN-PEG/TA 25 compared to control cells. Quantified data (Figure 7c) showed a significant reduction in the average number of FAs per cell (3.8 ± 1.7) with MSN-PEG/TA 25 treatment, compared to control cells (12 ± 2.8). These results indicated that MSN-PEG/TA 25 contributed to cell motility by influencing the remodeling of FA dynamics, leading to inhibition of cell migration. Given that cell migration depends on FA dynamics and rearrangement of the cytoskeleton via integrins,^47,48^ the structure of the cytoskeleton was also examined. However, no significant alterations were observed in the cytoskeleton (α-tubulin and F-actin) following MSN-PEG/TA 25 treatment (Figure S8a). Western blot results revealed no differences in the expression levels of G-actin or F-actin with MSN-PEG/TA 25 treatment (Figure S8b). Therefore, we concluded that MSN-PEG/TA 25 inhibits cell migration via a reduction in FA formation, not by rearrangement of the cytoskeleton.

To validate our hypothesis in vivo, we examined the expressions of p-paxillin, p-ERK, and p-FAK in the presence of MSN-PEG/TA 25 in tumor tissues using immunostaining (Figure 7d-f). Results showed that the levels of p-paxillin, p-ERK, and p-FAK (green) were significantly lower than those in the control groups, further verifying that MSN-PEG/TA 25 hinders tumor cell migration by impacting FA turnover. Similar results were observed in Western blot analyses (Figure 7g), where the expression of FAK-related proteins decreased.

**Figure 8:**
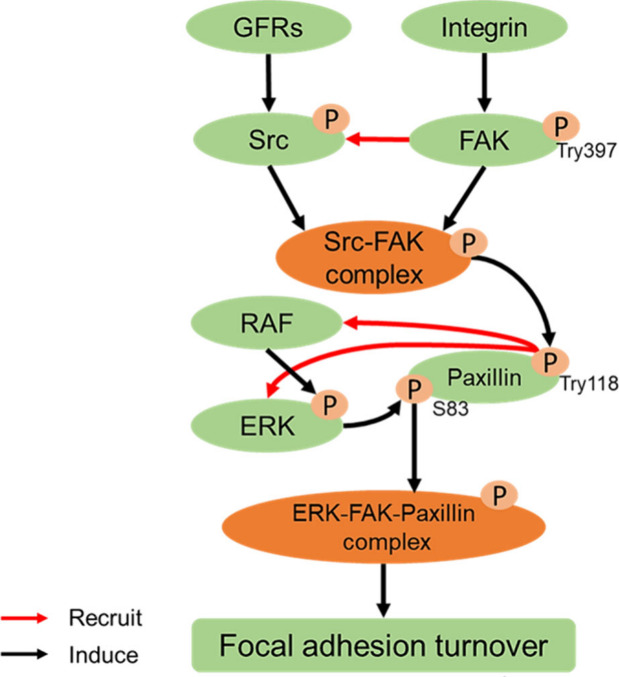
Pathway for focal adhesion turnover mediated by focal adhesion kinase (FAK), extracellular signal-regulated kinase (ERK), and paxillin.

Based on these results, we proposed a mechanism for how MSN-PEG/TA 25 disrupts the FAK-ERK-paxillin pathway (Figure 8), which is crucial in FA disassembly. In this mechanism, phosphorylation of FAK at Tyr397 recruits Src to compose the FAK-Src complex, which subsequently phosphorylates Tyr118 on paxillin to create an ERK-binding site, promoting recruitment of ERK and RAF. ERK phosphorylation of paxillin S83 enhances the recruitment of FAK to the paxillin scaffold, thus leading to FA disassembly.^49−52^ Overall, we demonstrated that MSN-PEG/TA 25 can interrupt the phosphorylation of FAK, ERK, and paxillin, further disrupting the FA turnover balance, followed by inhibition of cell motility. It was shown that gold nanorods could target cancer cell integrins, inhibiting cell migration by affecting cytoskeletal proteins.^53^ MSNs are known to disintegrate into sub-3 nm silica NPs in physiological media. It is anticipated that these small silica NPs can bind to integrins and modulate FAK-ERK-paxillin cytoskeletal proteins to inhibit cell migration.^54^ Previously, Whitehead and co-workers showed that silica NPs could increase intestinal permeability by binding to cell surface integrins, achieving enhanced oral drug delivery.^55^ Deng et al. reported that CuS@mSiO2-PEG NPs could inhibit tumor cell migration through a photothermal effect.^27^ Furthermore, Du et al. examined samples from 142 patients with cervical cancers and 20 normal cervix samples, finding that integrin α3 was highly expressed in patients and predicted poor overall survival and disease-free survival.^56^ They also showed that the integrin-FAK pathway could inhibit cell migration, proposing that α3 integrin as a candidate prognostic biomarker and therapeutic target in cancer patients. However, it is not clear whether CuS or silica were responsible for the effect on cell motility. Our findings suggest that silica-based NPs can inhibit cell migration via modulation of the FAK pathway and its subsequent signaling events.

Previous reports on NPs and cancer metastasis have highlighted the significant role of NP-induced endothelial leakiness (NanoEL) and thus increased metastasis effect instead.^57−59^ NanoEL refers to the disruption of the endothelial barrier by NPs, leading to increased blood vessel permeability. Setyawati et al. have found that NanoEL-induced endothelial leakiness can enhance migration.^57−59^ In seeming contradiction, we found that MSNs effectively inhibit cancer cell migration and endothelial tube formation (Figure 4). The explanation lies in the difference in the density of the NPs. Tay et al. highlighted that an effective density of silica NPs must exceed 1.57 g/cm^3^ to induce the NanoEL effect.^58^ Following the methods outlined in these papers, we calculated the density of our NPs and found that all MSN groups had a density lower than 1.50 g/cm^3^, whereas SSNs had a density close to 2.00 g/cm^3^ (Table S2). This suggests that MSNs are not able to induce NanoEL-related endothelial leakiness. Overall, our results indicate that MSN treatment reduces metastasis instead of NanoEL-induced leakage leading to increased metastasis.

### 3.8 MSN-PEG/TA 25 Modulates Angiogenesis in the Chick Chorioallantoic Membrane (CAM)

**Figure 9:**
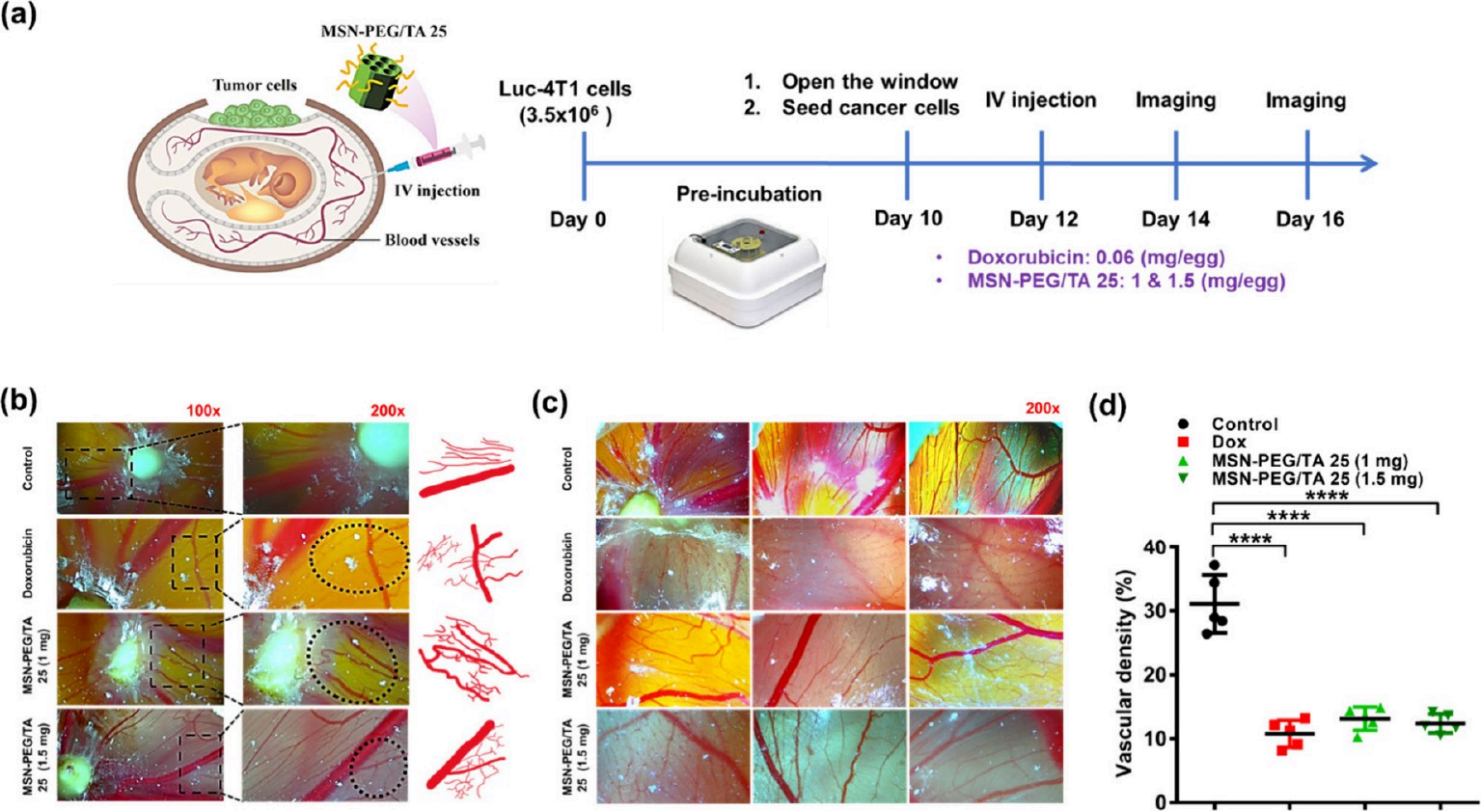
MSN-PEG/TA 25 inhibits angiogenesis in 4T1 xenograft chick chorioallantoic membrane (CAM). (a) CAM experimental scheme. 4T1 cells were seeded onto the CAM and Dox (0.06 mg/egg) or MSN-PEG/TA 25 (1 mg/egg or 1.5 mg/egg) was injected into the vein of the CAM 2 days postimplantation. Photographs of the CAM vasculature were captured on (b) days 14 and (c) 16 for angiogenesis analysis. (d) Statistical analysis of vascular density. Image analyses were performed using ImageJ software (**** *p* < 0.0001, *n* = 5).

Recent studies indicated that the FAK signaling cascade is important in tumor angiogenesis.^60^ Inhibiting FAK has been confirmed as an effective antiangiogenic therapeutic strategy for cancer treatment.^61^ Since MSNs can mitigate cell migration through interactions with the FAK pathway, MSNs can possibly be used to suppress tumor angiogenesis, as suggested by Figure 4c, where MSNs effectively inhibited tube formation—a critical step in angiogenesis. To further explore the influence of MSNs on tumor angiogenesis *in vivo*, a chicken embryo CAM tumor model was used for an angiogenesis analysis, as illustrated in Figure 9a. The CAM assay is widely used for cancer- and angiogenesis-related studies due to its unique properties, including easier and faster solid tumor formation, rapid vascular growth, a dense capillary network, and the *in vivo* environment.^62,63^

Figure 9b and Figure 9c depict the *in vivo* antiangiogenic effects of MSNs through the CAM assay. The control group, where CAM was implanted with a 4T1 tumor, displayed typical blood vessel growth patterns radiating toward the tumors. However, treatment with Dox (0.06 mg/egg) resulted in notable blood vessel disintegration, demonstrating a pronounced antiangiogenic effect^64,65^ Fewer vessels seemed to penetrate the tumors. Interestingly, MSN-PEG/TA 25 (1 mg/egg) disrupted regular vascularization patterns, significantly restricting tumor angiogenesis. Abnormal vascular growth was also noted at a higher dosage of MSN-PEG/TA 25 (1.5 mg/egg). Disruption of regular vascularization patterns, such as short, narrow, and disturbed irregular growth, was consistently documented in previous studies as an inhibitory phenomenon of tumor angiogenesis in the chick CAM model.^66−68^ The statistical analysis in Figure 9d and Figure S9 further confirmed that MSN-treated and Dox-treated groups demonstrated significantly diminished vascular densities compared to the control group. Similar to a report by Pastorino et al.,^66^ our results demonstrated that treatments with Dox or MSNs could decrease blood vessel density surrounding the xenograft. While consistent with Figure 1h, it is crucial to note that MSN treatment did not directly reduce the volume of xenograft tumors. Instead, it induced vascular anomalies and irregular growth, ultimately leading to irregular vessel density and impeded tumor metastasis. In short, our results underline that MSNs affect blood vessel growth and inhibit vascular endothelial cell migration, highlighting the potential of MSNs as a promising therapeutic strategy for antiangiogenesis applications.

## 4 Conclusions

Tumor metastasis is primarily driven by cell migration and angiogenesis, which are essential processes enabling the spread of cancer cells to distant organs. Unlike previous studies, our work highlights the unique porous structure and surface TA modification of MSN-type NPs (MSN-PEG/TA 25), conferring highly efficient antimetastatic activity without the need for drug loading. The basis of our approach is to target the focal adhesion pathway. At present, most of the fundamental studies on cancer metastasis have concentrated on EMT, and thus, many of the antimetastasis preclinical studies are based on the hypothesis of inhibiting EMT. However, EMT is a wide-ranging and complex pathway, making it a challenging target for antimetastatic therapies. Targeting EMT at the molecular level by blocking key transcription factors is pharmacologically challenging^69,70^ due to its timing and context sensitivity. Also, side effects are hard to avoid. On the other hand, targeting the focal adhesion pathway offers a more straightforward and less side-effect-prone strategy. Additionally, targeting the focal adhesion pathway is more effective because it directly inhibits cancer cell migration and disrupts metastasis at multiple stages (such as invasion and colonization), whereas targeting EMT only prevents the initial conversion of stationary cells into migratory ones. This makes targeting the focal adhesion pathway more effective than EMT modulation for halting metastasis.

We are the first to systematically investigate the molecular mechanisms, e.g. focal adhesion, behind the antimetastatic effects of MSN-PEG/TA 25, identifying the involvement of the focal adhesion turnover signaling pathway in modulating cell motility. Our findings suggest that MSNs can serve as effective antimetastatic nanocarriers with significant clinical potential for cancer therapy. While research on the antimetastatic effects of NPs remains limited, to the best of our knowledge, this study represents the first comprehensive exploration of MSN-PEG/TA’s antimetastatic capacity, particularly in an *in vivo* model. MSNs can serve as a platform technology for cancer therapy where the carried drugs can be changed according to the primary tumor type while at the same time the carrier itself can prevent the metastasis of the cancer. Overall, our results advance the field of cancer nanotherapeutics by providing insights into the molecular mechanisms linking MSNs to antitumor metastasis, emphasizing their therapeutic potential in cancer treatment.

## Data Availability

Data will be made available on request.

## Supporting Information Available

Antimetastatic activity, nitrogen adsorption isotherm, TGA analysis, NTA analysis, cell viability, antimetastasis-related gene expression levels, immunofluorescence assay of cytoskeleton staining, physiochemical characteristics of various NPs, quantitative analysis of vascular density (PDF)
MSN-PEG/TA 25 leakage from blood vessels (MP4)
3D live-cell imaging for control (AVI)
3D live-cell imaging for MSN-PEG/TA 25 (AVI)

## Supplementary Material

am4c16609_si_002.mp4

am4c16609_si_003.avi

am4c16609_si_004.avi
