# Supplementary material for: Drug-Free Mesoporous Silica Nanoparticles Enable Suppression of Cancer Metastasis and Confer Survival Advantages to Mice with Tumor Xenografts
Source: ACS Appl Mater Interfaces. 2024 Oct 24;16(45):61787–804. doi: 10.1021/acsami.4c16609 (PMC11565475; doi:10.1021/acsami.4c16609)
Supplement: Supplementary file 1 — am4c16609_si_001.pdf [file am4c16609_si_001.pdf]

# Supporting Information

# Drug-Free Mesoporous Silica Nanoparticles Enable Suppression of Cancer Metastasis and Confer Survival Advantages to Mice with Tumor Xenografts

Yu-Tse Lee[a†], Si-Han Wu[b,c†], Cheng-Hsun Wu[d], Yu-Han Lin[b], Cong-Kai Lin[e], Zih-An Chen[b,f], Ting-Chung Sun[d], Yin-Ju Chen[e], Peilin Chen[f], Chung-Yuan Mou[a,b] and Yi-Ping Chen[b,c]*

[a]Department of Chemistry, National Taiwan University, Taipei 10617, Taiwan

[b]Graduate Institute of Nanomedicine and Medical Engineering, College of Biomedical Engineering, Taipei Medical University, Taipei 11031, Taiwan

[c]International Ph.D. Program in Biomedical Engineering, College of Biomedical Engineering, Taipei Medical University, Taipei 11031, Taiwan

[d]Nano Targeting & Therapy Biopharma Inc., Taipei 10087, Taiwan

[e]Graduate Institute of Biomedical Materials & Tissue Engineering, College of Biomedical Engineering, Taipei Medical University, Taipei 11031, Taiwan

[f]Research Center for Applied Sciences, Academia Sinica, Taipei 11529, Taiwan

**† These authors contributed equally to this work.**

*** Correspondence: haychen@tmu.edu.tw**

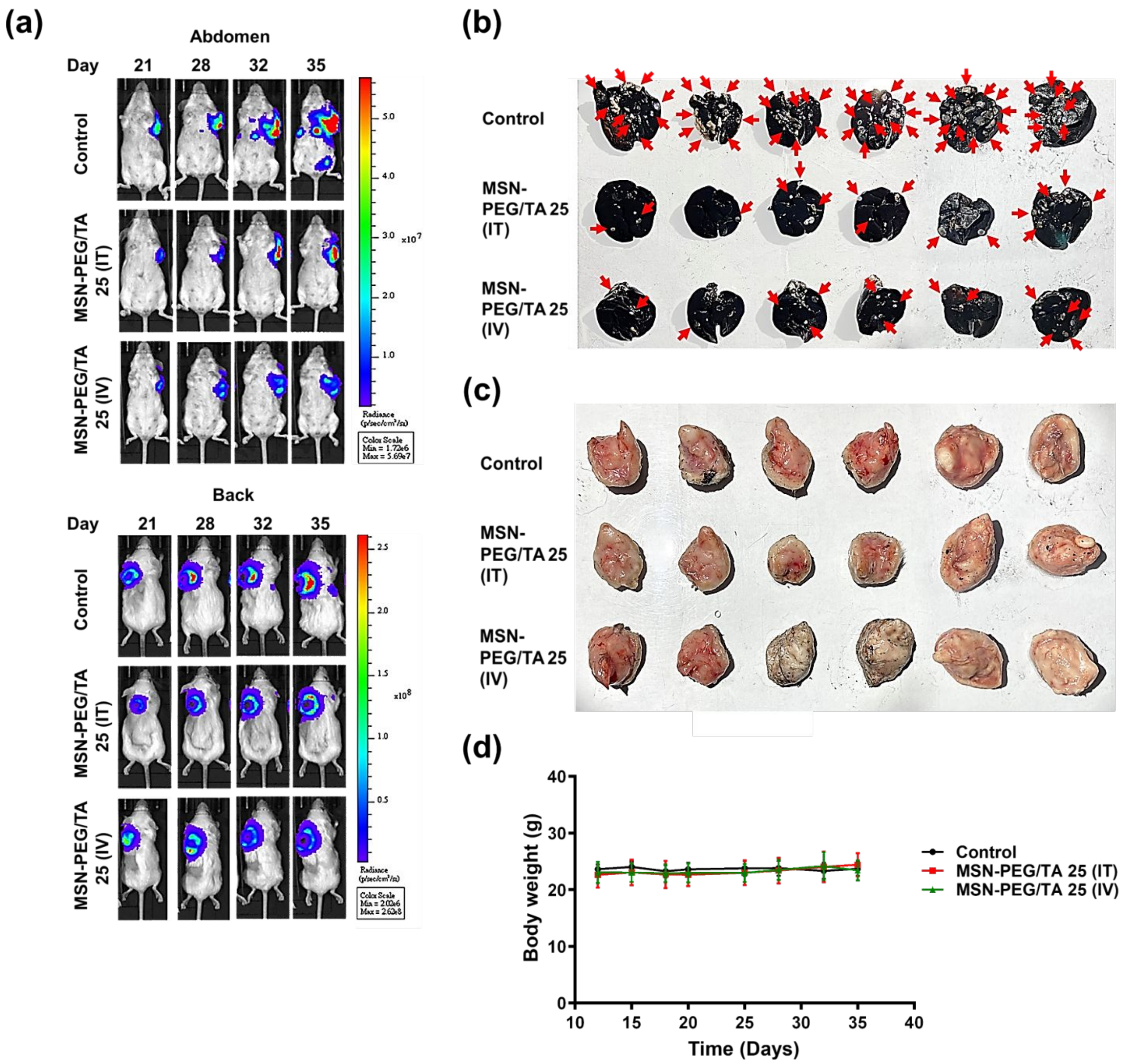


**Figure S1. Antimetastatic activity of MSN-PEG/TA 25 in mice with 4T1 xenografts.** Mice were implanted with $1.5\times10^6$ Luc-4T1 cells and then intratumorally (20 mg/kg) and intravenously (200 mg/kg) injected with MSN-PEG/TA 25 three times. (a) IVIS images of the tumor from days 21 to 35. (b) India ink-stained lung (back) on day 35. Red arrows point to metastatic nodules in the lung. (c) Tumor images and (d) body weight (n=6).

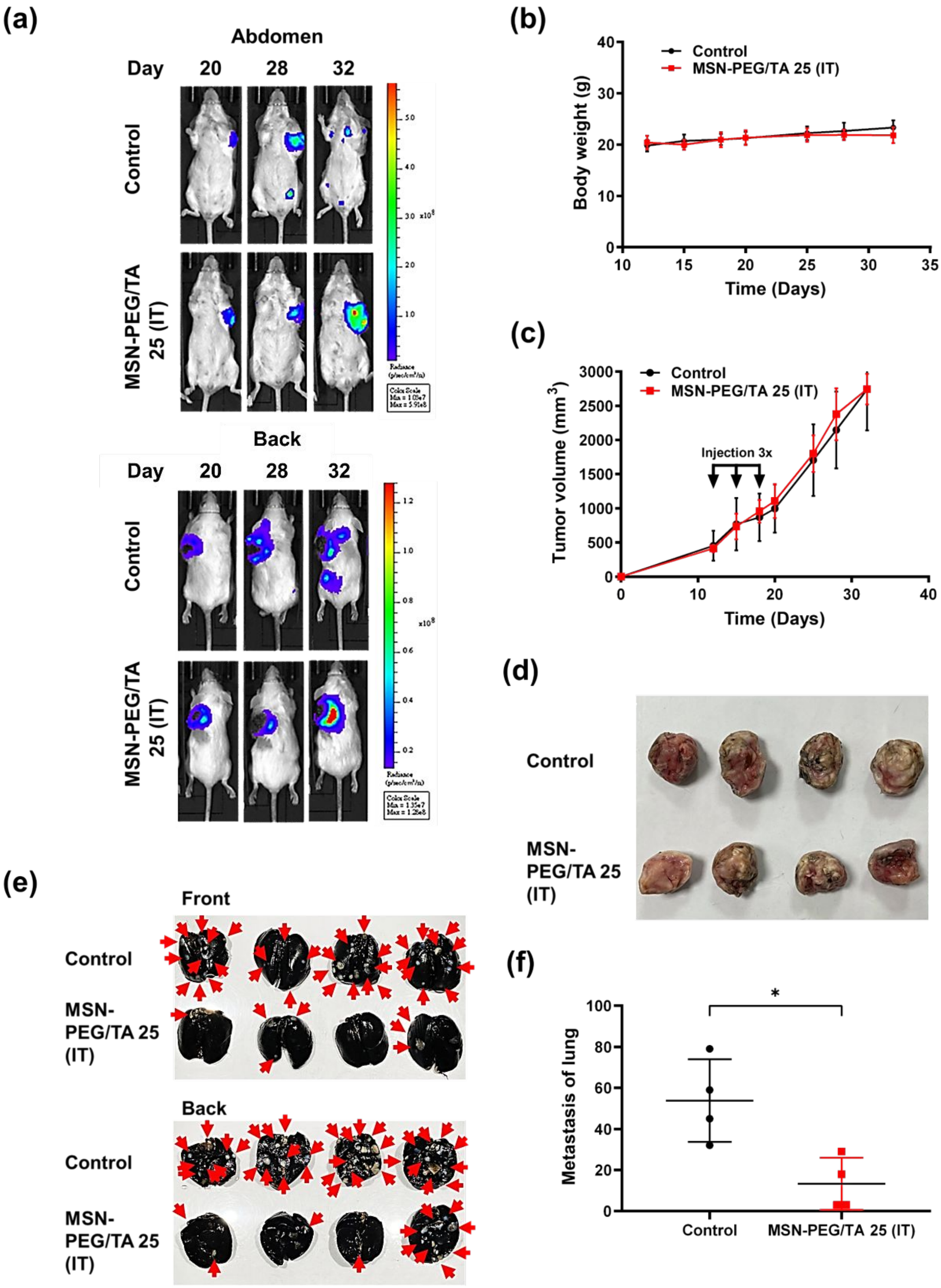


**Figure S2. Antimetastatic activity of MSN-PEG/TA 25 with a high concentration in mice with 4T1 xenografts.** Mice were implanted with $1.5\times10^6$ of Luc-4T1 cells and then intratumorally (200 mg/kg) injected with MSN-PEG/TA 25 three times. (a) IVIS image of the tumor. (b) Body weight. (c) Tumor volume. (d) Tumor images. (e) India ink-stained lung. Red arrows point to metastatic nodules in the lung. (f) Numbers of lung nodules with metastases. (* $p < 0.05$, n=4).

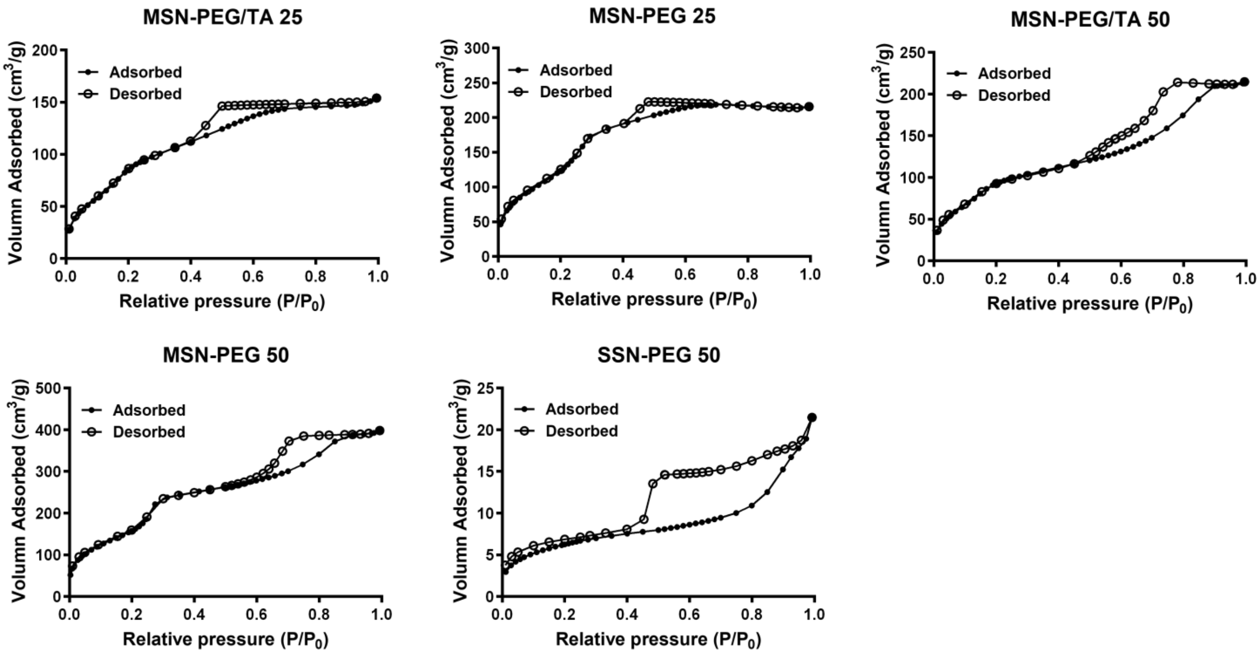


**Figure S3. Nitrogen adsorption isotherm of various types of silica nanoparticles.**

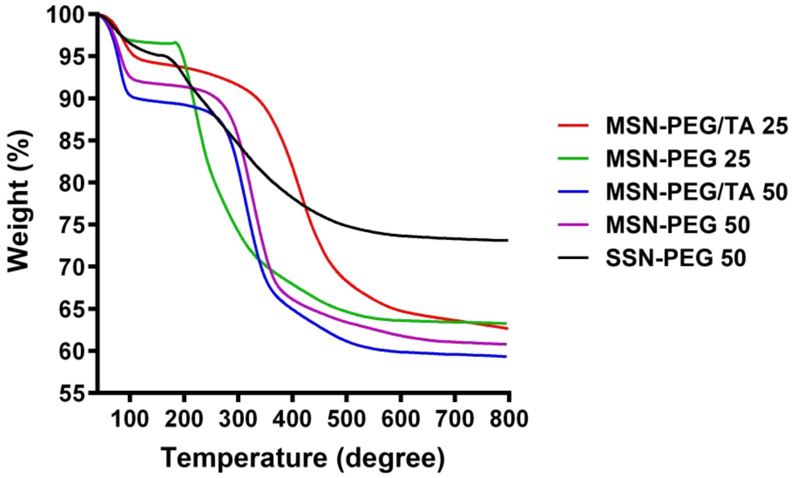


**Figure S4. Thermogravimetric analysis (TGA) of various types of silica nanoparticles.** Detailed results are described in Table S1.

**Table S1. Thermogravimetric analysis (TGA) results of various types of silica nanoparticles**

| Samples | TGA results for 40℃-200℃ (wt%) | TGA results for 200℃-500℃ (wt%) | TGA results for 500℃-800℃ (wt%) |
|---|---|---|---|
| MSN-PEG/TA 25 | 6.34% | 25.39% | 5.63% |
| MSN-PEG 25 | 5.56% | 29.79% | 1.39% |
| MSN-PEG/TA 50 | 10.76% | 28.09% | 1.81% |
| MSN-PEG 50 | 8.62% | 27.97% | 2.63% |
| SSN-PEG 50 | 7.35% | 17.80% | 1.75% |

wt%, normalized weight loss from the TGA.

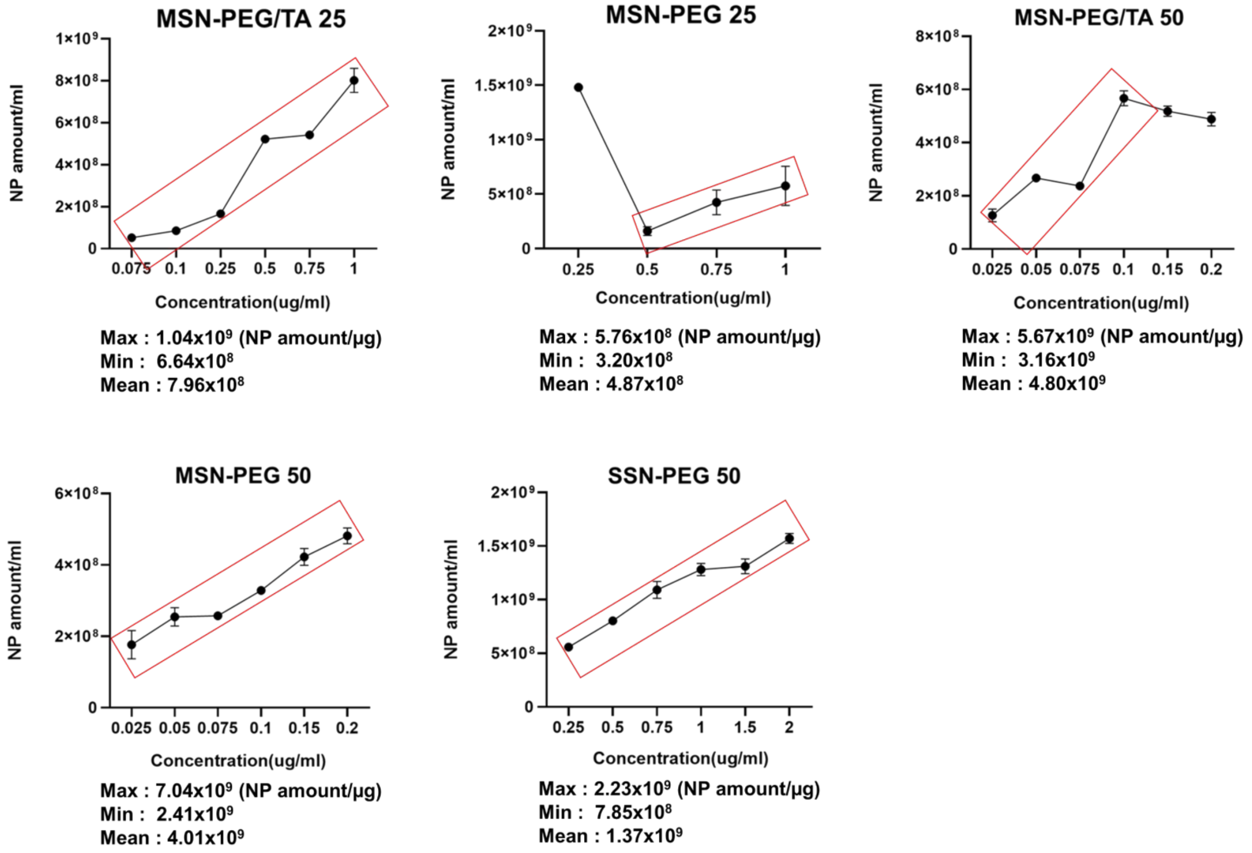


**Figure S5. Concentrations of various types of silica nanoparticles from a nanoparticle tracking analysis (NTA).**

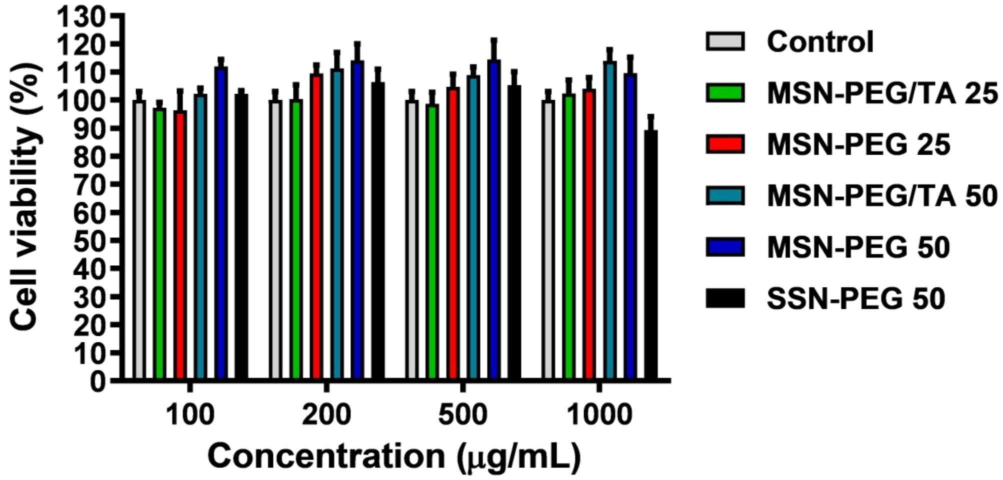


**Figure S6. Cell viability of various types of silica nanoparticles in 4T1 cells (n=3).**

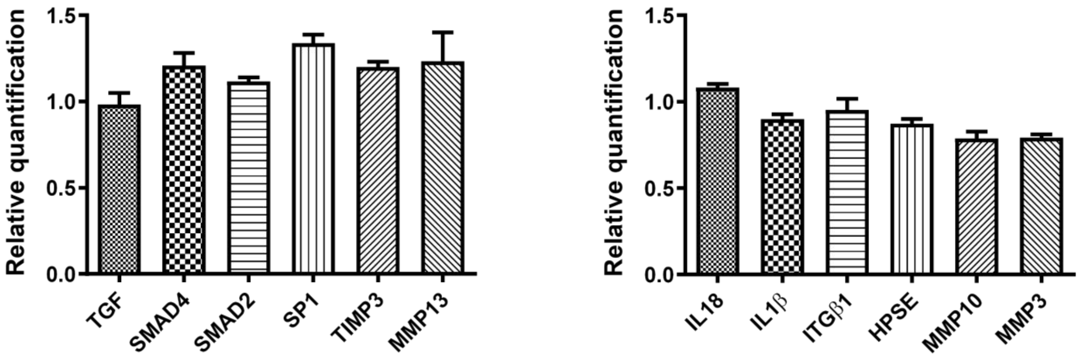


**Figure S7. Expression levels of various genes related to metastasis in 4T1 cells treated with MSN-PEG/TA 25 (n=3).**

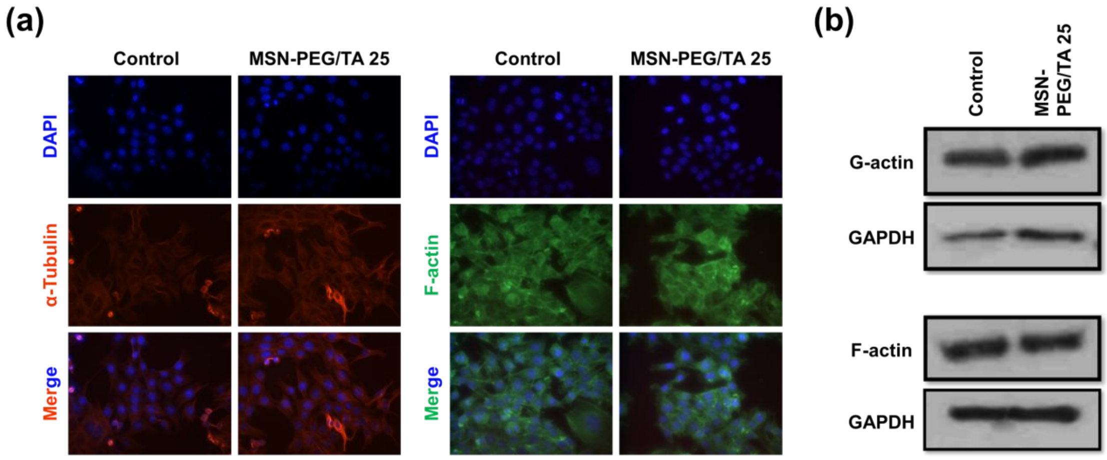


**Figure S8. Effect of MSN-PEG/TA 25 on the cytoskeleton.** (a) Immunofluorescence assay of cytoskeleton staining – α-tubulin (red), F-actin (green), and nuclei (blue). (b) Western blot analysis of G-actin and F-actin. GAPDH was used as a loading control.

**Table S2. Physiochemical characteristics of various types of silica nanoparticles**

| Sample | Diameter (nm) | Radius (nm) | Volume of particle ($cm^3$) | Internal Pore Volume ($cm^3$/ g) | Effective density ($\rho_{eff}$) (g / $cm^3$) |
|---|---|---|---|---|---|
| MSN-PEG/TA 25 | 26.4 | 13.2 | 0.010 | 0.54 | 1.46 |
| MSN-PEG 25 | 25.9 | 13.0 | 0.009 | 0.60 | 1.40 |
| MSN-PEG/TA 50 | 44.9 | 22.5 | 0.047 | 0.79 | 1.21 |
| MSN-PEG 50 | 45.4 | 22.7 | 0.049 | 0.88 | 1.12 |
| SSN-PEG 50 | 48.4 | 24.2 | 0.059 | 0.01 | 1.99 |

Diameter : counted from TEM;  Radius : 1/2 diameter; Volume of particle: 4/3 пr3;
Internal Pore Volume: obtained from BJH Adsorption dV/dD Pore Volume
The effective density ( $\rho_{eff}$ ) was calculated according to the following equations

$$\rho_{eff}=\frac{M_{total}-M_{pore}}{V_{total}}$$

where $M_{total}$ and $M_{pore}$ are the mass of the particle and internal pore, respectively.

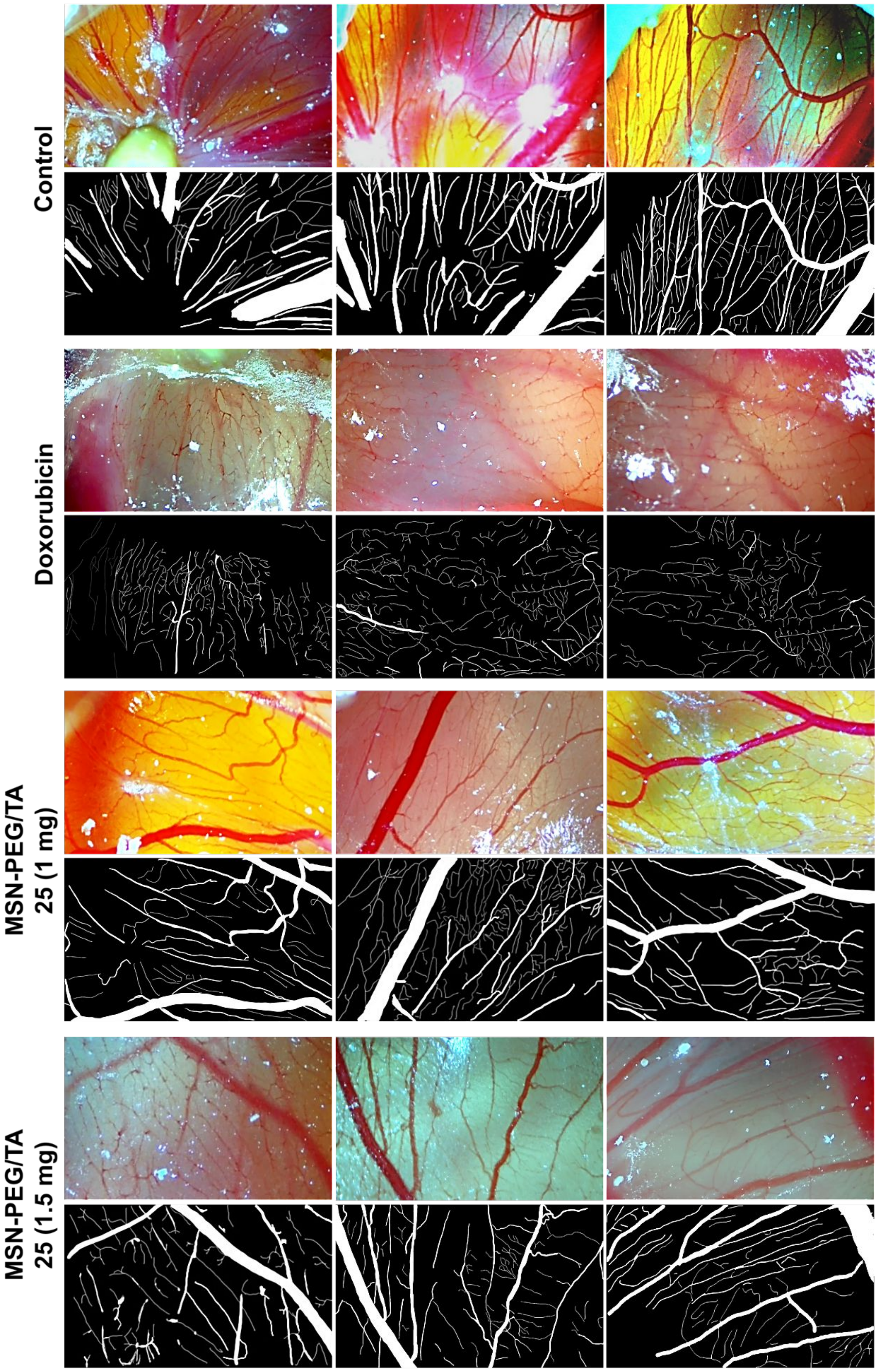


**Figure S9. The quantitative analysis of vascular density in the chick CAM.** Blood vessel densities were calculated by NIH ImageJ software with the "angiogenesis analyzer" plug-in.
